# Associations between Rest–Activity Rhythms and Liver Function Tests: The US National Health and Nutrition Examination Survey, 2011–2014

**DOI:** 10.3390/clockssleep5040045

**Published:** 2023-11-02

**Authors:** Chris Ho Ching Yeung, Cici Bauer, Qian Xiao

**Affiliations:** 1 Department of Epidemiology, Human Genetics and Environmental Sciences, School of Public Health, University of Texas Health Science Center at Houston, Houston, TX 77030, USA; 2 Department of Biostatistics and Data Science, School of Public Health, University of Texas Health Science Center at Houston, Houston, TX 77030, USA; cici.x.bauer@uth.tmc.edu; 3 Center for Spatial-Temporal Modeling for Applications in Population Sciences, School of Public Health, University of Texas Health Science Center at Houston, Houston, TX 77030, USA

**Keywords:** circadian rhythms, alkaline phosphatase, alanine transaminase, aspartate transaminase, gamma-glutamyl transferase

## Abstract

Liver functions are regulated by the circadian rhythm; however, whether a weakened circadian rhythm is associated with impaired liver function is unclear. This study aims to investigate the association of characteristics of rest–activity rhythms with abnormal levels of biomarkers of liver function. Data were obtained from the National Health and Nutrition Examination Survey 2011–2014. Seven rest–activity rhythm parameters were derived from 24 h actigraphy data using the extended cosine model and non-parametric methods. Multiple logistic regression and multiple linear regression models were used to assess the associations between rest–activity rhythm parameters and alanine aminotransferase (ALT), aspartate aminotransferase (AST), alkaline phosphatase (ALP), gamma-glutamyl transaminase (GGT), albumin and bilirubin. Weakened overall rhythmicity characterized by a lower F statistic was associated with higher odds of abnormally elevated ALP (OR_Q1vs.Q5_: 2.16; 95% CI 1.19, 3.90) and GGT (OR_Q1vs.Q5_: 2.04; 95% CI 1.30, 3.20) and abnormally lowered albumin (OR_Q1vs.Q5_: 5.15; 95% CI 2.14, 12.38). Similar results were found for a lower amplitude, amplitude:mesor ratio, interdaily stability and intradaily variability. Results were robust to the adjustment of confounders and cannot be fully explained by individual rest–activity behaviors, including sleep and physical activity. Weakened rest–activity rhythms were associated with worse liver function as measured by multiple biomarkers, supporting a potential role of circadian rhythms in liver health.

## 1. Introduction

Circadian rhythms refer to the 24 h cycle of physical, mental and behavioral fluctuations in the body orchestrated by the master circadian clock in the suprachiasmatic nucleus and numerous peripheral clocks throughout the body [1,2]. Weakened circadian function due to disruption or impairment of the internal biological clock (e.g., reduced output amplitude, abnormal phase/timing, irregular rhythmicity) has been linked to a wide range of adverse health effects, including cardiometabolic diseases and cancer [3,4,5]. In animal studies, mice with core circadian clock genes (e.g., *CLOCK*) mutations developed hyperlipidemia, hepatic steatosis and cancer [6,7]. The liver governs homeostasis of the body and plays a critical role in the metabolism of glucose, amino acids, proteins and lipids. Liver damage has been associated with a higher risk of diabetes and cardiovascular diseases [8,9]. Previous research reported that night shift work was associated with impaired liver function and liver diseases [10], suggesting that disrupted circadian rhythms may play a role in liver health. Such a connection is also supported by transcriptome analysis, demonstrating that most functions of the liver are regulated by the circadian rhythm [11] and suggesting that altered liver function due to weakened circadian rhythms may be a contributing factor to the association between circadian rhythms and metabolic diseases found in previous studies [12,13].

The rest–activity rhythm encompasses both the resting and active phases of human movements throughout the 24 h period. This behavioral cycle has a bidirectional relationship with the internal circadian rhythm [14]. It has been widely used as a proxy for circadian rhythms in large epidemiological studies because it is easier to measure and quantify. Studies have shown that characteristics of weakened rest–activity rhythms, such as impaired overall rhythmicity, lower amplitude, altered acrophase and higher intradaily variability, were associated with diabetes, cognitive impairment and cancer outcomes [12,15,16,17].

However, epidemiological evidence on the association of rest–activity rhythms with liver function and diseases is limited. Understanding the role of rest–activity rhythms in liver health may provide insights into improving liver functions and preventing liver and related cardiometabolic diseases via strategies aimed at improving circadian function and rest–activity patterns. To address this gap in the literature, we investigated the cross-sectional relationships between characteristics of rest–activity rhythms and levels of six biomarkers commonly assessed in the clinical setting, including four liver enzymes (i.e., alanine aminotransferase (ALT), aspartate aminotransferase (AST), alkaline phosphatase (ALP) and gamma-glutamyl transaminase (GGT)), albumin and bilirubin in a nationally representative sample of the US population from the National Health and Nutrition Examination Survey (NHANES) 2011–2014. Given the fact that the liver is extensively regulated by circadian rhythms and the growing evidence that circadian disruption is associated with adverse health effects, we hypothesized that weakened rest–activity rhythms are associated with worse liver function indicated by elevated liver enzyme and bilirubin levels and lowered albumin level.

## 2. Results

Demographic characteristics of the participants by quintiles of F statistic are presented in Table 1. Participants in the lower quintiles, reflecting a worse overall rhythmicity, were more likely to be men, non-Hispanic black, unmarried, have a lower household income, be a current smoker, obese, or diabetic, and have a lower total activity count and longer sleep duration.

**Figure 1:**
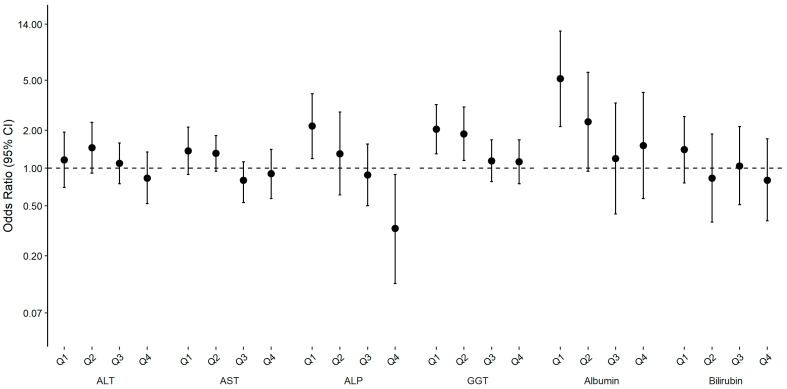
Associations (OR and 95% CI) between quintiles of F statistic and abnormal levels of liver enzymes, albumin and bilirubin, NHANES 2011–2014. The fifth quintile (Q5) acted as the reference group. Abnormal levels defined as >47 IU/L in men or >30 IU/L in women for ALT, >33 IU/L in men and women for AST, >113 IU/L in men and women for ALP, >65 IU/L in men or >36 IU/L in women for GGT, <3.7 g/dL in men and women for albumin, >1.3 mg/dL in men and women for bilirubin. Model adjusted for age, gender, race and ethnicity, education, household income, marital status, smoking, alcohol, hepatitis B, hepatitis C and hepatitis E. ALT, alanine aminotransferase; AST, aspartate aminotransferase; ALP, alkaline phosphatase; GGT, gamma-glutamyl transaminase; CI, confidence interval; OR, odds ratio.

Associations between the F statistic, the primary rest–activity rhythm parameter (i.e., overall rhythmicity or pseudo F statistic), and the likelihood of having abnormal liver enzyme, albumin and bilirubin levels are presented in Figure 1 and Table 2. In minimally adjusted models, weaker overall rhythmicity measured by the F statistic was associated with a higher likelihood of abnormal levels of AST, ALP, GGT and albumin (Model 1, Table 2). After further adjusting for sociodemographic factors, lifestyle factors, hepatitis status, diabetes status and BMI (Model 2–4, Table 2 and Figure 1), the results were attenuated, but the positive associations for ALP, GGT and albumin remained. In model 2, participants in the lowest quintile of F statistic were 2.16 (95% CI: 1.19–3.90) times, 2.04 (95% CI: 1.30–3.20) times and 5.15 (95% CI: 2.14–12.38) times more likely to have abnormal ALP, GGT and albumin levels, respectively, compared with participants in the highest quintile. For the analyses focusing on liver enzyme, albumin and bilirubin levels as continuous variables, lower F statistic quintiles were associated with higher levels of ALT, ALP and GGT and lower levels of albumin (Tables S2 and S3). After further adjusting for individual behavioral components of the rest–activity rhythm (i.e., total physical activity and sleep duration) (Table S4), the results became attenuated. However, the association between lower F statistic and higher odds of abnormal GGT (OR_Q1vs.Q5_, 95% CI: 1.78, 1.06–2.97) and albumin (OR_Q1vs.Q5_, 95% CI: 3.61, 1.38–9.46) remained. Similar associations were also found after removing night shift workers and/or participants with unconventional sleep timing (Table S5).

Results for additional rest–activity parameters are presented in Table 3, Table 4, Table 5, Table 6, Table 7 and Table 8. A lower amplitude was associated with higher odds of abnormal ALP (OR_Q1vs.Q5_, 95% CI: 2.66, 1.24–5.69), GGT (OR_Q1vs.Q5_, 95% CI: 1.82, 1.20–2.76) and albumin (OR_Q1vs.Q5_, 95% CI: 2.73, 1.20–6.19) (Table 3). A lower amplitude:mesor ratio was associated with higher odds of abnormal AST (OR_Q1vs.Q5_, 95% CI: 1.64, 1.02–2.62) and GGT (OR_Q1vs.Q5_, 95% CI: 1.75, 1.13–2.71) (Table 5). A lower IS was associated with higher odds of abnormal ALP (OR_Q1vs.Q5_, 95% CI: 2.56, 1.26–5.21), GGT (OR_Q1vs.Q5_, 95% CI: 1.72, 1.05–2.81) and albumin (OR_Q1vs.Q5_, 95% CI: 5.09, 1.94–13.39) (Table 7). A higher IV was associated with higher odds of abnormal ALP (OR_Q5vs.Q1_, 95% CI: 2.38, 1.20–4.71), albumin (OR_Q5vs.Q1_, 95% CI: 3.34, 1.05–10.68) and bilirubin (OR_Q4vs.Q1_, 95% CI: 4.05, 1.13–14.51) (Table 8). No association was found for mesor and acrophase after adjusting for multiple covariates in Model 2. In an analysis focusing on liver enzyme, albumin and bilirubin levels as continuous outcomes, similar results were found (Table S2). After further adjusting for total physical activity and sleep duration, the association between a lower amplitude:mesor ratio and higher odds of abnormal AST, a lower IS and higher odds of abnormal albumin and a high IV and higher odds of abnormal bilirubin remained (Table S4). 

Subgroup analyses were performed for the analyses of the four liver enzymes, stratified by alcohol intake, BMI, diabetes status and session attended (Tables S6–S9). Generally, results found across different subgroups of alcohol intake, BMI and session attended were similar to those in the overall sample with a wider CI, and we did not detect any interaction between rest–activity rhythm parameters and alcohol intake, BMI or session attended (Tables S6, S7 and S9). In the subgroup analysis by diabetes status, we found interactions with diabetes for the associations between amplitude and AST, mesor and ALP, amplitude:mesor ratio and AST, acrophase and GGT, and IV with ALP and GGT. In each of these cases, the association appeared stronger in effect sizes among people with diabetes. 

**Figure 2:**
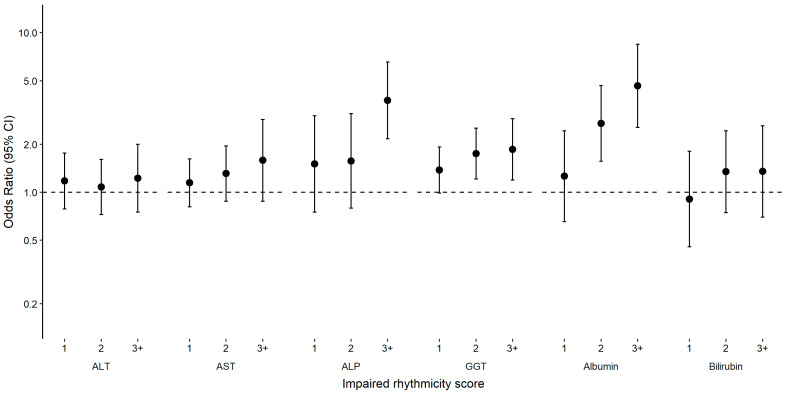
Associations (OR and 95% CI) between impaired rhythmicity score and abnormal levels of liver enzymes, albumin and bilirubin, NHANES 2011–2014. Impaired rhythmicity scores were derived for each participant by counting the number of rest–activity rhythm parameters in the quintile assumed to be the least healthy (Q1 for F statistic, amplitude and mesor, and Q5 for acrophase). The lowest score (0) acted as the reference group. Abnormal levels defined as >47 IU/L in men or >30 IU/L in women for ALT, >33 IU/L in men and women for AST, >113 IU/L in men and women for ALP, >65 IU/L in men or >36 IU/L in women for GGT, <3.7 g/dL in men and women for albumin, >1.3 mg/dL in men and women for bilirubin. Model adjusted for age, gender, race and ethnicity, education, household income, marital status, smoking, alcohol, hepatitis B, hepatitis C and hepatitis E. ALT, alanine aminotransferase; AST, aspartate aminotransferase; ALP, alkaline phosphatase; GGT, gamma-glutamyl transaminase; CI, confidence interval; OR, odds ratio.

Compared to the group with robust rhythmicity (i.e., composite score = 0), higher scores (1 to 3+) were associated with higher odds of abnormal ALP, GGT, and albumin, with a dose–response relationship (Figure 2 and Table S10). Moreover, participants in the lower quintile of F statistic, amplitude, mesor, amplitude:mesor ratio, and IS and higher quintile of IV were more likely to have an elevated composite score of abnormal liver enzymes (Table S11).

## 3. Discussion

In a nationally representative sample of noninstitutionalized US adults, we found weakened rest–activity rhythms characterized by a lower F statistic, amplitude, amplitude:mesor ratio, IS and IV were associated with higher odds of abnormally elevated liver enzyme levels, particularly for AST, ALP, GGT, and abnormally lowered albumin level. Participants with weaker overall rhythmicity, lower rhythm strength and lower average level of activity were also more likely to have multiple abnormal measures of liver enzymes, albumin and bilirubin. Results were robust to the adjustment of various confounders and removal of night shift workers and/or participants with unconventional sleep timing and cannot be fully explained by individual behaviors, including sleep and physical activity. 

Mounting evidence has linked rest–activity rhythm characteristics with various health outcomes, particularly metabolic dysfunction. For example, previous studies using similar rest–activity parameters showed that characteristics of a weakened rhythm were associated with higher fasting insulin and insulin resistance and higher odds of diabetes [12,13]. Although no epidemiological study directly assessed the association between rest–activity rhythms and liver enzymes, albumin and bilirubin, several studies suggested a role of circadian disruption in liver health. A study in 25 healthy adults showed that serum liver enzyme levels fluctuated with circadian rhythms, peaked around the evening and night, and bottomed in the early morning [18]. Another study in men showed that night shift workers had a higher risk of abnormally elevated ALT compared with day workers, with an increased dose response for longer years working the night shift [10]. In addition, previous studies, including meta-analyses, observed elevated liver enzymes and higher prevalence of hepatic steatosis and nonalcoholic steatohepatitis among individuals with nocturnal hypoxia and obstructive sleep apnea-hypopnea, which often lead to disrupted sleep [19,20,21,22]. Evidence from these studies, together with findings in our analysis, suggested that disruption in circadian rhythms may be associated with impaired liver function.

The exact mechanisms connecting circadian rhythms and liver enzymes and albumin levels remain unclear. However, growing evidence from mechanistic studies increasingly supports an important role of circadian rhythms in liver function. In animal studies, mouse liver showed an extensive circadian gene expression and circadian regulation of liver transcriptional cycles [23,24], suggesting liver function is at least partly regulated by the circadian clock. Higher risk of liver inflammation and hepatic steatosis were also observed in mice with genetically (*ClockΔ19* mutant) or environmentally disrupted circadian rhythms [7]. Similarly, sleep deprivation was found to induce hepatic steatosis and increase ALT and AST in mice [25,26]. Moreover, several studies identified multiple molecular pathways linking circadian rhythms with liver diseases. Brain and Muscle ARNT-Like 1 (*BMAL1*), which is a core clock gene, was found to be involved in the regulation of hepatic lipogenesis, and its overexpression was found to be protective against alcoholic liver damage in mice [27], supporting hepatoprotective effects of a robust circadian clock. Conversely, the circadian clock in the muscle was found to regulate daily rhythms of transcription of genes, including Malate dehydrogenase 1 (*MDH1*) and hydroxyacyl-CoA dehydrogenase trifunctional multienzyme complex subunit alpha (*HADHA*), which are involved in important liver functions such as the tricarboxylic acid cycle and fatty acid β-oxidation [28]. Such crosstalk between muscle and liver was proposed to be mediated through myokines released from muscle cells [29]. In addition, our sensitivity analyses showed the associations between weakened rest–activity rhythms and abnormal liver enzyme and albumin levels were partly attenuated after adjusting for BMI and diabetes status, and the associations were more prominent (i.e., larger effect size) in participants with diabetes, as shown in the stratified analysis. These results may suggest the associations were partly mediated by diabetes status or underlying factors associated with diabetes. This appears consistent with previous evidence that shows a weakened rest–activity rhythm is associated with a higher BMI and an increased risk of diabetes [12,30], as well as studies showing higher BMI and diabetes are associated with a higher risk of nonalcoholic fatty liver disease [31]. Together, these studies pinpointed multiple pathways, including lipogenesis, transcription regulation of key proteins involved in liver function, myokines, BMI and diabetes, that may underly the observed association between weakened circadian rhythms and abnormal levels of liver biomarkers. Future studies should clarify the mechanistic pathways linking circadian function to liver health in human populations. 

Of the various rest–activity parameters analyzed in our study, F statistic, amplitude, amplitude:mesor ratio, IS and IV showed associations with liver enzymes, while mesor and acrophase did not. These results may suggest the overall rest–activity rhythmicity (F statistic, IS and IV) and rhythm strength (amplitude and amplitude:mesor ratio) had stronger associations with liver enzyme levels when compared to the average level of activity (mesor) and the timing of activity (acrophase). Conversely, the dose–response relationship between a higher composite score of impaired rhythmicity and higher odds of having abnormal liver enzymes may support the utility of a composite measure of rest–activity rhythms as an indicator of liver function. Of the four liver enzymes, albumin and bilirubin, the results were more pronounced for AST, ALP, GGT and albumin. Although these are commonly used markers for liver function, they can also serve as indicators of damage in other organs, such as cancer and metabolic syndrome [32]. Future studies are needed to determine the underlying physiological changes and biological mechanisms that contribute to the observed association in the analysis and whether a higher risk of liver diseases is found in populations with weakened rest–activity rhythms. 

A major strength of our study is the inclusion of a large and nationally representative sample of US adults. Another strength is the use of actigraphy data to characterize rest–activity rhythms. Previous studies on circadian/rest–activity rhythms that relied on self-reported sleep quality and chronotype could be subjective and inaccurate. The availability of actigraphy data provides the possibility to objectively and more accurately measure 24 h rest–activity profiles and, in turn, allows us to derive meaningful parameters that are able to capture unique features of rest–activity rhythms. Moreover, NHANES also collected a large variety of information from participants, which allowed for a thorough analysis that included many confounders. The large sample size also made it possible to conduct subgroup analyses to assess any differences in the association between populations. The consistent results from different models and subgroups provided more robust evidence supporting the found association between rest–activity rhythms and liver function. 

Our study also has several limitations. First, the results were based on cross-sectional data and, hence, cannot be used to establish the temporal relationship and direction of associations between rest–activity rhythms and liver enzymes, albumin and bilirubin. Second, the rest–activity rhythm is only a proxy measure of the circadian rhythm. There is a need for future studies focusing on direct measures of the internal circadian clock, such as daily rhythms of melatonin and core body temperature. Third, rest–activity parameters were derived from the extended cosine model, which assumed a cosine-like pattern of the rest–activity rhythm. This assumption may not apply to certain participants with more severe circadian disruptions. Fourth, the small sample size in certain subgroups with abnormal liver enzyme levels limited the statistical power in detecting an association. Fifth, the levels of the liver enzymes, albumin and bilirubin may also be affected by health conditions other than liver diseases [32]. Further studies are needed to investigate whether a higher risk of liver diseases is found in populations with weakened rest–activity rhythms. Finally, given the nature of an observational study, residual confounding may still exist even after adjusting for various potential confounders. Therefore, replicating the analysis and triangulating the results from other populations and applying other analytical methods possessing different strengths and limitations (e.g., Mendelian randomization) could provide us with additional evidence towards establishing the association between rest–activity rhythms and liver functions. 

To conclude, our study supports an association between weakened rest–activity rhythms and higher odds of abnormally elevated liver enzymes and abnormally lowered albumin, thus contributing to the growing literature linking adverse health outcomes with weakened circadian function. Future studies should focus on examining prospective associations connecting rest–activity rhythms and liver functions and elucidating the underlying mechanisms. 

## 4. Methods

### 4.1. Study Population

Data were obtained from the 2011–2014 NHANES cross-sectional survey [33]. The objective of NHANES is to assess the health and nutritional status of all US adults and children in a nationally representative sample of the civilian noninstitutionalized population [33]. The NHANES interview collected information on demographic, socioeconomic, dietary and other lifestyle factors and a wide range of health outcomes. It also performed medical, dental and physiological measurements as well as laboratory tests for biomarkers [33]. The National Center for Health Statistics Research Ethics Review Board reviewed and approved the NHANES study [34].

### 4.2. Measurement of Rest–Activity Rhythms

All participants aged three years and older were asked to wear a physical activity monitor (ActiGraph model GT3X+) on the non-dominant wrist for seven consecutive days to collect 24 h movement data [35,36]. The physical activity monitor measured triaxial acceleration every 1/80 of a second, and the values were summed over each minute [35,36]. The quality of data collected was reviewed by contractors at Northeastern University in Boston under the direction of collaborators and staff from the National Cancer Institute and the National Center for Health Statistics. The summary data for each 1 min epoch were then used to classify the epoch as wake, sleep, non-wear or unknown, using a published algorithm [35]. In this analysis, a valid day of measurement was defined as having at least 20 h of wake and sleep data. We only included participants with at least 4 valid days of measurement.

Parametric and non-parametric rest–activity rhythm parameters were derived from the 5 min average of triaxial acceleration activity data. Parametric parameters were derived using the extended cosine model [37]. Activity data were fit to a squared waveform by applying an anti-log transformation. The primary rest–activity parameter of interest was the pseudo F statistic, a goodness-of-fit measure to quantify the overall rhythmicity [37]. Higher values of F statistic suggest more robust overall rest–activity rhythmicity. Four other parameters of the rest–activity rhythm were also examined, including (1) amplitude, calculated as the difference between the peak and nadir of the fitted activity curve—higher values of amplitude represent stronger rhythms; (2) mesor, calculated as the sum of minimum and half of the amplitude, representing the average level of activity; (3) amplitude:mesor ratio, representing a normalized measure of rhythm strength accounting for the average activity level; and (4) acrophase, representing the time of peak activity, with a higher value representing a later peak. In addition, non-parametric parameters, including interdaily stability (IS) and intradaily variability (IV), were also derived to assess the strength and disturbance of rest–activity rhythm. The seven rest–activity rhythm parameters could be classified into four categories, including (1) overall rhythmicity (F statistic, IS and IV), (2) rhythm strength (amplitude and amplitude:mesor ratio), (3) average level of activity (mesor), and (4) timing of activity (acrophase). The correlation matrix of the rest–activity rhythm parameters is presented in Table S1. All parameters were grouped by quintiles, with the reference quintile presumed to have the lowest risk of abnormal liver enzymes, albumin and bilirubin (Q1 for acrophase and IV, Q5 for the other five parameters).

We derived a composite score of impaired rhythmicity by counting the number of rest–activity rhythm parameters in the quintile assumed to be the least healthy (Q1 for F statistic, amplitude and mesor, and Q5 for acrophase), with 0 representing robust rhythmicity and 3+ representing the most impaired rhythmicity.

### 4.3. Measurement of Liver Enzymes, Albumin and Bilirubin

We chose six measurements of liver function, including ALT, ASP, AST, GGT, albumin and bilirubin, because they are widely and regularly assessed in a clinical setting and are available in the NHANES [38].

Serum specimens were collected from participants throughout the day. These specimens were stored under appropriate frozen (−30 °C) conditions and shipped to the National Center for Environmental Health for testing [39]. Kinetic and enzymatic rate methods were used to measure serum liver enzymes (ALT, ASLP, AST and GGT) levels using Beckman UniCel^®^ DxC800 Synchron [39]. Albumin and bilirubin levels were measured with a bichromatic digital endpoint method and a timed-endpoint Diazo method, respectively [39]. Levels beyond the normal values indicated in the NHANES Laboratory Procedure Manual were defined as abnormal (ALT: >47 IU/L in men, >30 IU/L in women, AST: >33 IU/L, ALP: >113 IU/L, GGT: >65 IU/L in men, >36 IU/L in women, albumin: <3.7 g/dL, bilirubin: >1.3 mg/dL) in this analysis [40]. We also derived a composite score of abnormal liver enzymes. This score (0 to 3+) was assigned to each participant based on the number of abnormal liver enzymes, albumin levels and bilirubin levels observed.

### 4.4. Covariates

In the analysis, we included multiple potential confounders based on the criterion that these variables may be associated with rest–activity rhythms and a cause of alterations of liver enzymes but are not on the causal pathway mediating the effect of rest–activity rhythms on liver enzyme levels [41]. These variables included participant’s age (continuous), gender (male, female), race and ethnicity (non-Hispanic white, non-Hispanic black, Hispanic, others), education level (less than high school, high school graduate, some college, college graduate or above), household income (<$20 k, $20 k–44.9 k, $45 k–74.9 k, $75 k+), marital status (married, not married), and body mass index (BMI, <25, 25–30, 30+). We also included self-reported smoking status (never smoker or less than 100 cigarettes in life, former smoker, current smoker), alcohol consumption levels (never or former drinker, light drinker (average daily drinking volume <1 for men and <0.5 for women), moderate drinker (1 to 2 for men and 0.5 to 1 for women), heavy drinker (≥2 for men and ≥1 for women)), and diabetes status (yes, no) determined by self-report or laboratory blood test of glycohemoglobin level, fasting glucose level and oral glucose tolerance test. Other liver-related diseases included the infection status of hepatitis B (hepatitis B core antibody or surface antigen positivity), hepatitis C (hepatitis C antibody or RNA positive) and hepatitis E (hepatitis E IgG or IgM antibody positive) determined by the laboratory blood test. Sleep duration and total physical activity were derived from the actigraphy data and included as covariates to assess to what degree the association observed for rest–activity rhythms is explained by individual behavioral components in the 24 h cycle [36]. BMI, diabetes status and alcohol drinking status were used to define subgroups in stratified analyses.

### 4.5. Analytic Sample

NHANES 2011–2014 included a total of 19,931 participants. We focused on adults aged 20 or above (*N* = 11,329). We additionally excluded participants who were 80 or older (*N* = 715), as all participants aged above 79 were coded as 80 years due to disclosure risk, which prevented us from obtaining the exact age for this age group [42]. Pregnant women were also excluded (*N* = 122). Of the remaining participants (*N* = 10,492), we further excluded those with no actigraphy data (*N* = 2513), with less than four days of valid actigraphy data (*N* = 1253), and those without liver enzyme, albumin and bilirubin measures (*N* = 344). The final analytic sample consisted of 6382 participants.

### 4.6. Statistical Analysis

NHANES full sample mobile examination center exam weight was applied to all analyses to account for the sampling design in the NHANES. To assess the association of rest–activity rhythm characteristics with odds of having abnormal liver enzyme, albumin or bilirubin levels, multiple logistic regression was performed, and the results were presented in odds ratio (OR) and 95% confidence interval (CI). To model liver enzyme, albumin and bilirubin levels as continuous outcome variables, we log-transformed the biomarker levels to improve normality, and then performed multiple linear regression and presented the beta coefficients and 95% CI. For the analysis focusing on the composite score of abnormal liver enzymes, we used ordinal logistic regression and presented the results in OR and 95% CI. In model 1, we adjusted for age and gender. In model 2 (main model), we further adjusted for race and ethnicity, education, household income, marital status, smoking status and alcohol consumption levels, as well as infection status of hepatitis B, hepatitis C and hepatitis E. In addition to model 2, we further adjusted for diabetes status in model 3 and BMI in model 4. In the sensitivity analysis, we further adjusted for individual behavior components of the rest–activity rhythm, including total physical activity and sleep duration, to examine to what degree these factors explain the association between rest–activity characteristics and liver enzyme levels. To test for trend, quintiles of rest–activity rhythm parameters were modeled as continuous, and statistical significance was assessed by the Wald test. In the subgroup analysis, we stratified by alcohol intake, BMI and diabetes status, as they are important risk factors for liver disease and may modify the association between rest–activity rhythms and liver function. In addition, we also stratified the analysis by blood sample collection session attended (morning, afternoon, evening), as the level of liver enzymes, albumin and bilirubin may vary at different times of day. Interaction between rest–activity rhythm parameters and alcohol intake, BMI, diabetes status and session attended was tested by the likelihood ratio test comparing a model with the cross-product term to one without. As data on shift work status is not available, we used two standard deviations away from the median midpoint of L5 (which indicates the midpoint in time of the 5 consecutive hours of the day with the lowest activity) as an indicator of night shift work and/or unconventional sleep timing. In the sensitivity analysis, we assessed the association between F statistic and liver enzymes after removing these participants to assess the potential impact of the inclusion of night shift workers and/or people with unconventional sleep timing, who may have a different rest–activity rhythm. Analyses were also performed using the impaired rhythmicity score, which reflects the extent of worsened rest–activity rhythm in different areas. This study followed the STROBE cross-sectional reporting guidelines [43]. Subgroup analyses were not performed for albumin and bilirubin due to small sample sizes in some subgroups. All analyses were performed in R (version 4.1.2).

## Figures and Tables

**Table 1:** Demographic characteristics of included participants from NHANES 2011–2014 by quintiles of F statistic.

	F Statistic ^a^					*p* Value ^b^
	Q1	Q2	Q3	Q4	Q5	
Age, median (IQR)	48 (33, 60)	50 (34, 60)	47 (34, 59)	48 (36, 62)	50 (37, 60)	0.11
Women, %	44	51	49	54	57	<0.001
Race, %						<0.001
Non-Hispanic white	60	63	67	70	74	
Non-Hispanic black	18	14	11	9	4	
Hispanic	14	14	15	15	16	
Others	8	10	8	6	7	
Education, %						<0.001
Less than high school	15	16	14	15	16	
High school graduate	21	20	22	20	22	
Some college	40	36	31	29	27	
College graduate or above	24	27	33	36	35	
Household Income, %						<0.001
<$20 k	22	20	14	12	11	
$20–$44.9 k	31	27	25	23	24	
$45–$74.9 k	17	21	21	19	23	
>$75 k	30	32	41	45	41	
Married, %	42	51	59	61	68	<0.001
Smoking, %						<0.001
Never	47	53	58	60	61	
Former	21	24	22	28	25	
Current	32	23	20	13	14	
BMI, %						<0.001
<25	22	25	25	28	36	
25–30	29	31	34	37	35	
≥30	48	45	41	36	29	
Diabetes, %	21	18	14	12	9	<0.001
Alcohol, %						0.3
Never/Former	30	28	25	25	23	
Light	52	55	54	53	55	
Moderate	9.4	8.9	10	11	11	
Heavy	9.2	8	11	11	11	
Hepatitis B core antibody or surface antigen positive, %	5.5	5.7	5.0	4.6	3.7	0.12
Hepatitis C antibody (confirmed) or RNA positive, %	4.0	1.9	1.6	0.8	0.8	<0.001
Hepatitis E IgG or IgM positive, %	7.7	6.2	6.6	6.4	8.6	0.40
Total activity count, median (IQR) ^c^	9253 (7279, 11,709)	10,105 (8455, 11,992)	10,995 (9477, 12,664)	11,567 (9961, 13,384)	12,996 (11,262, 14,724)	<0.001
Sleep duration (minutes), median (IQR)	411 (357, 492)	415 (365, 477)	406 (364, 454)	408 (364, 449)	402 (362, 441)	<0.001
Shift workers (proxied by L5MD), %	16	4.3	2.1	2.4	2	<0.001

Values were weighted using sample weights. ^a^ Higher F statistic indicates stronger overall rhythmicity. ^b^ *p*-values were derived from Chi-square test for categorical variables and Kruskal–Wallis test for continuous variables. ^c^ Measured as the total daily sum of the Monitor-Independent Movement Summary triaxial value. Abbreviations: NHANES, National Health and Nutrition Examination Survey; IQR, interquartile range.

**Table 2:** Associations (OR and 95% CI) between quintiles of F statistic and abnormal levels of liver enzymes, albumin and bilirubin, NHANES 2011–2014.

Abnormal Levels of Liver Enzymes ^a^	F Statistic					*p* Trend
	Q1	Q2	Q3	Q4	Q5	
F statistic, median (IQR)	181 (128–225)	325 (295–354)	453 (420–481)	599 (560–650)	851 (764–997)	
ALT						
N (%) ^b^	148 (11)	146 (13)	138 (11)	102 (8)	124 (9.8)	
OR (95% CI)						
Model 1	1.21 (0.83, 1.75)	1.37 (0.95, 1.98)	1.1 (0.81, 1.49)	0.8 (0.55, 1.18)	ref.	0.02
Model 2	1.16 (0.7, 1.93)	1.45 (0.91, 2.31)	1.09 (0.75, 1.58)	0.83 (0.52, 1.34)	ref.	0.08
Model 3	1.06 (0.62, 1.81)	1.36 (0.84, 2.2)	1.03 (0.7, 1.52)	0.8 (0.5, 1.29)	ref.	0.20
Model 4	1.02 (0.57, 1.82)	1.32 (0.82, 2.12)	1 (0.67, 1.48)	0.79 (0.48, 1.31)	ref.	0.26
AST						
N (%) ^b^	184 (14)	157 (11)	115 (8.2)	103 (8.4)	104 (9)	
OR (95% CI)						
Model 1	1.55 (1.18, 2.03)	1.27 (0.99, 1.63)	0.86 (0.63, 1.19)	0.91 (0.66, 1.26)	ref.	<0.001
Model 2	1.37 (0.89, 2.12)	1.31 (0.94, 1.81)	0.8 (0.53, 1.23)	0.9 (0.57, 1.41)	ref.	0.04
Model 3	1.26 (0.8, 1.99)	1.24 (0.88, 1.75)	0.77 (0.5, 1.18)	0.87 (0.56, 1.37)	ref.	0.10
Model 4	1.32 (0.82, 2.14)	1.26 (0.89, 1.8)	0.79 (0.5, 1.23)	0.88 (0.55, 1.42)	ref.	0.07
ALP						
N (%) ^b^	78 (5.8)	45 (3.5)	31 (2.1)	21 (0.9)	32 (2.4)	
OR (95% CI)						
Model 1	2.82 (1.7, 4.69)	1.52 (0.77, 3.01)	0.98 (0.62, 1.54)	0.37 (0.18, 0.78)	ref.	<0.001
Model 2	2.16 (1.19, 3.9)	1.3 (0.61, 2.79)	0.88 (0.5, 1.55)	0.33 (0.12, 0.89)	ref.	0.002
Model 3	1.87 (1.02, 3.4)	1.17 (0.54, 2.55)	0.81 (0.46, 1.44)	0.3 (0.11, 0.83)	ref.	0.004
Model 4	2.12 (1.09, 4.15)	1.21 (0.54, 2.69)	0.83 (0.45, 1.53)	0.26 (0.09, 0.76)	ref.	0.004
GGT						
N (%) ^b^	178 (13)	148 (12)	130 (7.9)	106 (7.6)	88 (7.1)	
OR (95% CI)						
Model 1	2.24 (1.63, 3.07)	1.9 (1.29, 2.79)	1.22 (0.9, 1.66)	1.11 (0.8, 1.54)	ref.	<0.001
Model 2	2.04 (1.3, 3.2)	1.87 (1.15, 3.06)	1.14 (0.78, 1.68)	1.12 (0.75, 1.68)	ref.	0.003
Model 3	1.85 (1.18, 2.91)	1.75 (1.05, 2.89)	1.08 (0.73, 1.6)	1.07 (0.73, 1.59)	ref.	0.01
Model 4	1.75 (1.05, 2.93)	1.66 (0.97, 2.84)	1.02 (0.68, 1.55)	1.05 (0.69, 1.61)	ref.	0.02
Albumin						
N (%) ^b^	89 (6.1)	40 (2.8)	29 (1.5)	28 (2.1)	11 (1)	
OR (95% CI)						
Model 1	7.25 (3.4, 15.48)	2.95 (1.34, 6.49)	1.56 (0.66, 3.73)	2.13 (0.94, 4.84)	ref.	<0.001
Model 2	5.15 (2.14, 12.38)	2.33 (0.94, 5.78)	1.19 (0.43, 3.3)	1.51 (0.57, 4.01)	ref.	0.001
Model 3	4.64 (1.87, 11.53)	2.12 (0.82, 5.49)	1.1 (0.37, 3.28)	1.45 (0.54, 3.87)	ref.	0.002
Model 4	4.28 (1.65, 11.1)	1.85 (0.67, 5.15)	1.01 (0.33, 3.13)	1.35 (0.47, 3.88)	ref.	0.003
Bilirubin						
N (%) ^b^	47 (4)	26 (2.3)	32 (3)	34 (2.5)	30 (2.5)	
OR (95% CI)						
Model 1	1.37 (0.85, 2.22)	0.84 (0.42, 1.7)	1.05 (0.57, 1.95)	0.92 (0.48, 1.77)	ref.	0.34
Model 2	1.4 (0.76, 2.58)	0.83 (0.37, 1.87)	1.04 (0.51, 2.14)	0.8 (0.38, 1.71)	ref.	0.34
Model 3	1.38 (0.73, 2.61)	0.82 (0.36, 1.85)	1.03 (0.49, 2.17)	0.8 (0.37, 1.74)	ref.	0.36
Model 4	1.51 (0.77, 2.96)	0.88 (0.39, 2.02)	1.12 (0.5, 2.49)	0.85 (0.37, 1.96)	ref.	0.24

^a^ Defined as >47 IU/L in men or >30 IU/L in women for ALT, >33 IU/L for AST, >113 IU/L for ALP, >65 IU/L in men or >36 IU/L in women for GGT, <3.7 g/dL for albumin, and >1.3 mg/dL for bilirubin. ^b^ Percentage weighted using sample weights. Model 1: adjusted for age and gender. Model 2: adjusted for age, gender, race and ethnicity, education, household income, marital status, smoking, alcohol, hepatitis B, hepatitis C and hepatitis E. Model 3: adjusted for age, gender, race and ethnicity, education, household income, marital status, smoking, alcohol, hepatitis B, hepatitis C, hepatitis E and diabetes status. Model 4: adjusted for age, gender, race and ethnicity, education, household income, marital status, smoking, alcohol, hepatitis B, hepatitis C, hepatitis E and BMI. Abbreviations: NHANES, National Health and Nutrition Examination Survey; ALT, alanine aminotransferase; AST, aspartate aminotransferase; ALP, alkaline phosphatase; GGT, gamma-glutamyl transaminase; IQR, interquartile range; CI, confidence interval; OR, odds ratio.

**Table 3:** Associations (OR and 95% CI) between quintiles of amplitude and abnormal levels of liver enzymes, albumin and bilirubin, NHANES 2011–2014.

Abnormal Levels of Liver Enzymes ^a^	Amplitude					*p* Trend
	Q1	Q2	Q3	Q4	Q5	
Amplitude, median (IQR)	4.6 (3.7–5.2)	6.7 (6.3–7.2)	8.5 (8.1–9.0)	10.8 (10.1–11.6)	14.7 (13.4–16.9)	
ALT						
N (%) ^b^	131 (11)	153 (12)	123 (9.7)	121 (8.8)	130 (11)	
OR (95% CI)						
Model 1	1.14 (0.81, 1.60)	1.24 (0.85, 1.82)	0.91 (0.63, 1.32)	0.79 (0.49, 1.29)	ref.	0.07
Model 2	1.30 (0.84, 2.01)	1.47 (0.97, 2.23)	1.09 (0.70, 1.69)	0.93 (0.51, 1.69)	ref.	0.02
Model 3	1.17 (0.73, 1.87)	1.38 (0.88, 2.16)	1.06 (0.67, 1.68)	0.92 (0.49, 1.72)	ref.	0.08
Model 4	1.18 (0.75, 1.86)	1.38 (0.88, 2.14)	1.06 (0.66, 1.69)	0.9 (0.48, 1.7)	ref.	0.06
AST						
N (%) ^b^	166 (13)	142 (10)	124 (9.4)	110 (7.4)	121 (11)	
OR (95% CI)						
Model 1	1.21 (0.79, 1.84)	0.95 (0.67, 1.37)	0.90 (0.62, 1.30)	0.69 (0.44, 1.09)	ref.	0.20
Model 2	1.34 (0.78, 2.31)	1.09 (0.70, 1.70)	1.07 (0.65, 1.74)	0.78 (0.41, 1.46)	ref.	0.13
Model 3	1.23 (0.69, 2.18)	1.04 (0.64, 1.67)	1.05 (0.63, 1.74)	0.77 (0.4, 1.49)	ref.	0.24
Model 4	1.31 (0.73, 2.37)	1.07 (0.66, 1.71)	1.06 (0.63, 1.79)	0.77 (0.4, 1.51)	ref.	0.16
ALP						
N (%) ^b^	75 (5.4)	39 (2.7)	28 (1.3)	35 (3.2)	30 (1.9)	
OR (95% CI)						
Model 1	2.47 (1.49, 4.10)	1.22 (0.69, 2.16)	0.59 (0.28, 1.24)	1.51 (0.85, 2.68)	ref.	0.004
Model 2	2.66 (1.24, 5.69)	1.54 (0.71, 3.34)	0.84 (0.32, 2.21)	2.21 (0.96, 5.07)	ref.	0.03
Model 3	2.35 (1.08, 5.12)	1.39 (0.62, 3.12)	0.78 (0.28, 2.13)	2.18 (0.93, 5.08)	ref.	0.07
Model 4	2.46 (1.09, 5.58)	1.48 (0.65, 3.37)	0.75 (0.27, 2.09)	2.13 (0.88, 5.18)	ref.	0.05
GGT						
N (%) ^b^	179 (13)	144 (10)	106 (8.3)	110 (8.1)	111 (7.7)	
OR (95% CI)						
Model 1	1.67 (1.21, 2.31)	1.29 (0.96, 1.73)	0.97 (0.66, 1.42)	0.95 (0.63, 1.43)	ref.	<0.001
Model 2	1.82 (1.20, 2.76)	1.50 (1.04, 2.15)	1.23 (0.77, 1.96)	1.18 (0.73, 1.89)	ref.	0.004
Model 3	1.65 (1.08, 2.53)	1.4 (0.95, 2.07)	1.19 (0.73, 1.94)	1.17 (0.71, 1.92)	ref.	0.02
Model 4	1.65 (1.05, 2.58)	1.37 (0.92, 2.04)	1.18 (0.72, 1.95)	1.14 (0.68, 1.92)	ref.	0.02
Albumin						
N (%) ^b^	88 (6)	35 (2.3)	27 (1.4)	21 (1.8)	26 (1.7)	
OR (95% CI)						
Model 1	3.88 (1.96, 7.7)	1.35 (0.62, 2.94)	0.78 (0.32, 1.92)	0.96 (0.39, 2.36)	ref.	0.001
Model 2	2.73 (1.2, 6.19)	1.13 (0.46, 2.74)	0.66 (0.23, 1.88)	0.72 (0.26, 1.99)	ref.	0.01
Model 3	2.48 (1.04, 5.92)	1.05 (0.41, 2.69)	0.63 (0.2, 1.95)	0.71 (0.25, 2.02)	ref.	0.03
Model 4	2.25 (0.95, 5.32)	0.99 (0.39, 2.5)	0.6 (0.2, 1.83)	0.67 (0.23, 1.9)	ref.	0.04
Bilirubin						
N (%) ^b^	38 (2.9)	24 (1.5)	34 (3.3)	36 (3.6)	37 (2.5)	
OR (95% CI)						
Model 1	1.38 (0.79, 2.43)	0.7 (0.29, 1.66)	1.58 (0.86, 2.93)	1.73 (0.89, 3.33)	ref.	0.78
Model 2	1.31 (0.63, 2.72)	0.69 (0.23, 2.11)	1.59 (0.7, 3.59)	1.79 (0.79, 4.06)	ref.	0.51
Model 3	1.29 (0.63, 2.65)	0.68 (0.22, 2.15)	1.58 (0.68, 3.67)	1.78 (0.77, 4.12)	ref.	0.46
Model 4	1.35 (0.63, 2.89)	0.7 (0.21, 2.3)	1.57 (0.65, 3.77)	1.77 (0.74, 4.28)	ref.	0.59

^a^ Defined as >47 IU/L in men or >30 IU/L in women for ALT, >33 IU/L for AST, >113 IU/L for ALP, >65 IU/L in men or >36 IU/L in women for GGT, <3.7 g/dL for albumin, and >1.3 mg/dL for bilirubin. ^b^ Percentage weighted using sample weights. Model 1: adjusted for age and gender. Model 2: adjusted for age, gender, race and ethnicity, education, household income, marital status, smoking, alcohol, hepatitis B, hepatitis C and hepatitis E. Model 3: adjusted for age, gender, race and ethnicity, education, household income, marital status, smoking, alcohol, hepatitis B, hepatitis C, hepatitis E and diabetes status. Model 4: adjusted for age, gender, race and ethnicity, education, household income, marital status, smoking, alcohol, hepatitis B, hepatitis C, hepatitis E and BMI. Abbreviations: NHANES, National Health and Nutrition Examination Survey; ALT, alanine aminotransferase; AST, aspartate aminotransferase; ALP, alkaline phosphatase; GGT, gamma-glutamyl transaminase; IQR, interquartile range; CI, confidence interval; OR, odds ratio.

**Table 4:** Associations (OR and 95% CI) between quintiles of mesor and abnormal levels of liver enzymes, albumin and bilirubin, NHANES 2011–2014.

Abnormal Levels of Liver Enzymes ^a^	Mesor					*p* Trend
	Q1	Q2	Q3	Q4	Q5	
Mesor, median (IQR)	3.1 (2.9–3.3)	3.6 (3.5–3.7)	4.0 (3.9–4.1)	4.4 (4.3–4.6)	5.2 (4.9–5.6)	
ALT						
N (%) ^b^	143 (11)	117 (9)	139 (11)	128 (10)	131 (11)	
OR (95% CI)						
Model 1	1.17 (0.87, 1.59)	0.92 (0.67, 1.28)	1.15 (0.92, 1.45)	0.99 (0.74, 1.33)	ref.	0.49
Model 2	1.33 (0.87, 2.05)	1.04 (0.65, 1.67)	1.36 (0.96, 1.92)	1.12 (0.76, 1.66)	ref.	0.27
Model 3	1.24 (0.78, 1.97)	1 (0.6, 1.65)	1.32 (0.92, 1.9)	1.1 (0.73, 1.67)	ref.	0.49
Model 4	1.2 (0.75, 1.93)	0.99 (0.6, 1.65)	1.31 (0.89, 1.91)	1.14 (0.74, 1.76)	ref.	0.63
AST						
N (%) ^b^	154 (11)	112 (7.9)	139 (11)	126 (10)	132 (10)	
OR (95% CI)						
Model 1	1.02 (0.75, 1.38)	0.71 (0.49, 1.04)	1.03 (0.75, 1.42)	1.02 (0.68, 1.52)	ref.	0.31
Model 2	1.21 (0.79, 1.84)	0.84 (0.53, 1.34)	1.27 (0.82, 1.96)	1.12 (0.67, 1.86)	ref.	0.79
Model 3	1.14 (0.73, 1.77)	0.82 (0.5, 1.33)	1.25 (0.79, 1.96)	1.11 (0.65, 1.88)	ref.	0.91
Model 4	1.16 (0.74, 1.82)	0.83 (0.5, 1.36)	1.25 (0.79, 1.99)	1.13 (0.65, 1.95)	ref.	0.99
ALP						
N (%) ^b^	69 (5)	32 (2)	29 (1.9)	30 (1.8)	47 (3.5)	
OR (95% CI)						
Model 1	1.26 (0.83, 1.93)	0.51 (0.26, 1.02)	0.49 (0.28, 0.84)	0.45 (0.24, 0.84)	ref.	0.13
Model 2	1.47 (0.79, 2.72)	0.69 (0.28, 1.69)	0.65 (0.35, 1.24)	0.50 (0.21, 1.22)	ref.	0.08
Model 3	1.29 (0.67, 2.48)	0.64 (0.26, 1.56)	0.62 (0.32, 1.23)	0.48 (0.19, 1.22)	ref.	0.16
Model 4	1.4 (0.68, 2.87)	0.66 (0.25, 1.74)	0.66 (0.33, 1.33)	0.51 (0.2, 1.33)	ref.	0.15
GGT						
N (%) ^b^	146 (11)	119 (7.4)	131 (9.6)	132 (8.9)	122 (9.7)	
OR (95% CI)						
Model 1	1.12 (0.79, 1.58)	0.73 (0.54, 0.97)	0.96 (0.7, 1.30)	0.85 (0.61, 1.19)	ref.	0.77
Model 2	1.33 (0.89, 2.00)	0.89 (0.62, 1.29)	1.12 (0.76, 1.67)	0.91 (0.62, 1.32)	ref.	0.20
Model 3	1.22 (0.8, 1.85)	0.85 (0.58, 1.26)	1.08 (0.72, 1.63)	0.88 (0.59, 1.31)	ref.	0.37
Model 4	1.18 (0.75, 1.87)	0.84 (0.58, 1.24)	1.06 (0.71, 1.59)	0.91 (0.61, 1.37)	ref.	0.54
Albumin						
N (%) ^b^	60 (3.6)	40 (2.7)	37 (2.5)	33 (2.1)	27 (1.6)	
OR (95% CI)						
Model 1	2.43 (1.15, 5.13)	1.74 (0.69, 4.37)	1.63 (0.76, 3.5)	1.24 (0.61, 2.52)	ref.	0.03
Model 2	2.22 (0.95, 5.18)	1.81 (0.63, 5.14)	1.73 (0.76, 3.95)	1.2 (0.52, 2.76)	ref.	0.06
Model 3	1.99 (0.83, 4.77)	1.71 (0.58, 5.07)	1.65 (0.72, 3.81)	1.17 (0.51, 2.72)	ref.	0.10
Model 4	1.8 (0.71, 4.59)	1.62 (0.52, 5.02)	1.58 (0.65, 3.86)	1.18 (0.5, 2.79)	ref.	0.18
Bilirubin						
N (%) ^b^	34 (3)	37 (3.2)	26 (1.5)	31 (3)	41 (3.5)	
OR (95% CI)						
Model 1	0.96 (0.54, 1.69)	0.99 (0.59, 1.67)	0.46 (0.22, 0.95)	0.97 (0.49, 1.92)	ref.	0.92
Model 2	0.81 (0.4, 1.67)	0.98 (0.51, 1.86)	0.43 (0.17, 1.08)	1.03 (0.45, 2.34)	ref.	0.56
Model 3	0.81 (0.38, 1.69)	0.97 (0.5, 1.88)	0.43 (0.17, 1.1)	1.03 (0.44, 2.38)	ref.	0.54
Model 4	0.86 (0.39, 1.89)	0.99 (0.48, 2.05)	0.44 (0.17, 1.16)	1.02 (0.43, 2.45)	ref.	0.72

^a^ Defined as >47 IU/L in men or >30 IU/L in women for ALT, >33 IU/L for AST, >113 IU/L for ALP, >65 IU/L in men or >36 IU/L in women for GGT, <3.7 g/dL for albumin, and >1.3 mg/dL for bilirubin. ^b^ Percentage weighted using sample weights. Model 1: adjusted for age and gender. Model 2: adjusted for age, gender, race and ethnicity, education, household income, marital status, smoking, alcohol, hepatitis B, hepatitis C and hepatitis E. Model 3: adjusted for age, gender, race and ethnicity, education, household income, marital status, smoking, alcohol, hepatitis B, hepatitis C, hepatitis E and diabetes status. Model 4: adjusted for age, gender, race and ethnicity, education, household income, marital status, smoking, alcohol, hepatitis B, hepatitis C, hepatitis E and BMI. Abbreviations: NHANES, National Health and Nutrition Examination Survey; ALT, alanine aminotransferase; AST, aspartate aminotransferase; ALP, alkaline phosphatase; GGT, gamma-glutamyl transaminase; IQR, interquartile range; CI, confidence interval; OR, odds ratio.

**Table 5:** Associations (OR and 95% CI) between quintiles of amplitude:mesor and abnormal levels of liver enzymes, albumin and bilirubin, NHANES 2011–2014.

Abnormal Levels of Liver Enzymes ^a^	Amplitude: Mesor					*p* Trend
	Q1	Q2	Q3	Q4	Q5	
Amplitude:Mesor, median (IQR)	1.1 (0.9–1.3)	1.7 (1.6–1.8)	2.1 (2.0–2.3)	2.8 (2.6–3.0)	3.7 (3.5–4.0)	
ALT						
N (%) ^b^	145 (12)	128 (10)	124 (9.8)	134 (10)	127 (10)	
OR (95% CI)						
Model 1	1.31 (0.93, 1.85)	1.07 (0.77, 1.48)	0.97 (0.67, 1.41)	0.98 (0.70, 1.37)	ref.	0.12
Model 2	1.45 (0.95, 2.23)	1.19 (0.81, 1.75)	1.05 (0.67, 1.64)	1.02 (0.62, 1.67)	ref.	0.05
Model 3	1.34 (0.85, 2.11)	1.14 (0.76, 1.72)	1.02 (0.64, 1.62)	1.01 (0.6, 1.69)	ref.	0.13
Model 4	1.38 (0.86, 2.22)	1.18 (0.79, 1.76)	1.04 (0.64, 1.69)	1.03 (0.6, 1.75)	ref.	0.08
AST						
N (%) ^b^	186 (15)	116 (8.6)	109 (8.2)	123 (9)	129 (11)	
OR (95% CI)						
Model 1	1.48 (0.98, 2.24)	0.82 (0.54, 1.23)	0.80 (0.50, 1.28)	0.88 (0.65, 1.20)	ref.	0.17
Model 2	1.64 (1.02, 2.62)	0.93 (0.59, 1.48)	0.87 (0.54, 1.40)	0.99 (0.67, 1.47)	ref.	0.10
Model 3	1.53 (0.93, 2.52)	0.92 (0.56, 1.49)	0.85 (0.52, 1.38)	0.99 (0.65, 1.5)	ref.	0.18
Model 4	1.64 (0.99, 2.72)	0.92 (0.57, 1.51)	0.87 (0.53, 1.44)	0.99 (0.65, 1.51)	ref.	0.11
ALP						
N (%) ^b^	68 (4.7)	44 (3.3)	33 (2.1)	34 (2.4)	28 (1.9)	
OR (95% CI)						
Model 1	2.21 (1.19, 4.10)	1.58 (0.90, 2.77)	0.95 (0.46, 1.95)	1.15 (0.53, 2.49)	ref.	0.01
Model 2	2.07 (0.88, 4.85)	1.82 (0.89, 3.72)	1.25 (0.52, 3.03)	1.45 (0.50, 4.14)	ref.	0.03
Model 3	1.93 (0.82, 4.51)	1.74 (0.82, 3.69)	1.22 (0.5, 2.97)	1.43 (0.47, 4.31)	ref.	0.046
Model 4	2.06 (0.84, 5.02)	1.74 (0.8, 3.78)	1.18 (0.45, 3.06)	1.44 (0.47, 4.43)	ref.	0.04
GGT						
N (%) ^b^	193 (15)	117 (8)	112 (8.3)	109 (8.3)	119 (8.3)	
OR (95% CI)						
Model 1	1.77 (1.23, 2.55)	0.88 (0.63, 1.22)	0.87 (0.61, 1.22)	0.90 (0.61, 1.32)	ref.	0.01
Model 2	1.75 (1.13, 2.71)	0.92 (0.60, 1.39)	1.08 (0.71, 1.64)	0.95 (0.58, 1.55)	ref.	0.02
Model 3	1.63 (1.04, 2.55)	0.88 (0.57, 1.36)	1.05 (0.67, 1.64)	0.94 (0.57, 1.57)	ref.	0.04
Model 4	1.68 (1.03, 2.73)	0.91 (0.57, 1.45)	1.07 (0.68, 1.69)	0.96 (0.56, 1.64)	ref.	0.04
Albumin						
N (%) ^b^	82 (5.8)	30 (2)	30 (1.9)	21 (1.5)	34 (2.1)	
OR (95% CI)						
Model 1	2.67 (1.41, 5.08)	0.87 (0.44, 1.72)	0.77 (0.36, 1.64)	0.62 (0.32, 1.2)	ref.	0.01
Model 2	1.99 (0.86, 4.59)	0.76 (0.34, 1.71)	0.69 (0.29, 1.66)	0.56 (0.26, 1.17)	ref.	0.09
Model 3	1.84 (0.76, 4.47)	0.73 (0.31, 1.73)	0.67 (0.27, 1.69)	0.54 (0.25, 1.17)	ref.	0.12
Model 4	1.83 (0.75, 4.46)	0.66 (0.26, 1.68)	0.68 (0.27, 1.71)	0.54 (0.24, 1.22)	ref.	0.14
Bilirubin						
N (%) ^b^	36 (3)	27 (2.2)	36 (2.6)	40 (3.9)	30 (2.3)	
OR (95% CI)						
Model 1	1.56 (0.85, 2.88)	1.12 (0.52, 2.39)	1.46 (0.81, 2.64)	2.09 (1.03, 4.24)	ref.	0.76
Model 2	1.47 (0.64, 3.38)	1.09 (0.42, 2.82)	1.4 (0.65, 3.02)	1.9 (0.73, 4.92)	ref.	0.92
Model 3	1.45 (0.63, 3.37)	1.08 (0.41, 2.88)	1.4 (0.63, 3.08)	1.89 (0.71, 5.04)	ref.	0.95
Model 4	1.49 (0.61, 3.64)	1.1 (0.39, 3.08)	1.35 (0.6, 3.04)	1.91 (0.68, 5.32)	ref.	0.89

^a^ Defined as >47 IU/L in men or >30 IU/L in women for ALT, >33 IU/L for AST, >113 IU/L for ALP, >65 IU/L in men or >36 IU/L in women for GGT, <3.7 g/dL for albumin, and >1.3 mg/dL for bilirubin. ^b^ Percentage weighted using sample weights. Model 1: adjusted for age and gender. Model 2: adjusted for age, gender, race and ethnicity, education, household income, marital status, smoking, alcohol, hepatitis B, hepatitis C and hepatitis E. Model 3: adjusted for age, gender, race and ethnicity, education, household income, marital status, smoking, alcohol, hepatitis B, hepatitis C, hepatitis E and diabetes status. Model 4: adjusted for age, gender, race and ethnicity, education, household income, marital status, smoking, alcohol, hepatitis B, hepatitis C, hepatitis E and BMI. Abbreviations: NHANES, National Health and Nutrition Examination Survey; ALT, alanine aminotransferase; AST, aspartate aminotransferase; ALP, alkaline phosphatase; GGT, gamma-glutamyl transaminase; IQR, interquartile range; CI, confidence interval; OR, odds ratio.

**Table 6:** Associations (OR and 95% CI) between quintiles of acrophase and abnormal levels of liver enzymes, albumin and bilirubin, NHANES 2011–2014.

Abnormal Levels of Liver Enzymes ^a^	Acrophase					*p* Trend
	Q1	Q2	Q3	Q4	Q5	
Acrophase, median (IQR)	13.3 (12.8–13.7)	14.3 (14.1–14.4)	14.9 (14.7–15.0)	15.6 (15.4–15.9)	17.2 (16.6–18.0)	
ALT						
N (%) ^b^	122 (9.7)	119 (10)	136 (10)	142 (11)	139 (11)	
OR (95% CI)						
Model 1	ref.	0.97 (0.67, 1.41)	0.96 (0.65, 1.42)	1.03 (0.71, 1.49)	0.99 (0.65, 1.51)	0.92
Model 2	ref.	1.02 (0.65, 1.58)	0.9 (0.58, 1.42)	0.96 (0.62, 1.48)	0.95 (0.59, 1.55)	0.73
Model 3	ref.	1.03 (0.64, 1.65)	0.91 (0.57, 1.45)	0.94 (0.61, 1.46)	0.93 (0.56, 1.55)	0.61
Model 4	ref.	1.03 (0.65, 1.65)	0.92 (0.57, 1.5)	0.99 (0.62, 1.58)	0.94 (0.56, 1.57)	0.71
AST						
N (%) ^b^	134 (11)	123 (9.3)	128 (9.3)	133 (10)	145 (11)	
OR (95% CI)						
Model 1	ref.	0.93 (0.61, 1.43)	0.95 (0.69, 1.31)	1.04 (0.72, 1.5)	1.13 (0.74, 1.71)	0.41
Model 2	ref.	0.99 (0.59, 1.67)	0.95 (0.63, 1.42)	0.98 (0.65, 1.47)	1.07 (0.67, 1.7)	0.81
Model 3	ref.	0.98 (0.57, 1.67)	0.94 (0.62, 1.42)	0.96 (0.64, 1.43)	1.03 (0.64, 1.66)	0.97
Model 4	ref.	0.99 (0.57, 1.73)	0.95 (0.62, 1.47)	0.98 (0.63, 1.52)	1.06 (0.66, 1.71)	0.81
ALP						
N (%) ^b^	52 (3.6)	35 (3.2)	32 (1.9)	36 (2.1)	52 (3.1)	
OR (95% CI)						
Model 1	ref.	0.87 (0.48, 1.58)	0.53 (0.27, 1.03)	0.62 (0.35, 1.11)	1.03 (0.69, 1.55)	0.34
Model 2	ref.	1.07 (0.54, 2.13)	0.68 (0.29, 1.59)	0.66 (0.33, 1.33)	0.99 (0.61, 1.62)	0.31
Model 3	ref.	1.09 (0.55, 2.16)	0.69 (0.3, 1.59)	0.66 (0.32, 1.37)	0.95 (0.58, 1.56)	0.25
Model 4	ref.	1.08 (0.52, 2.24)	0.71 (0.29, 1.71)	0.61 (0.28, 1.3)	0.94 (0.52, 1.71)	0.24
GGT						
N (%) ^b^	122 (9.2)	122 (8.6)	123 (8.6)	120 (9.1)	163 (11)	
OR (95% CI)						
Model 1	ref.	0.87 (0.61, 1.26)	0.89 (0.59, 1.35)	0.98 (0.68, 1.41)	1.34 (0.94, 1.91)	0.08
Model 2	ref.	0.97 (0.62, 1.53)	0.96 (0.57, 1.62)	0.89 (0.56, 1.41)	1.2 (0.79, 1.82)	0.52
Model 3	ref.	0.99 (0.61, 1.59)	0.97 (0.56, 1.68)	0.88 (0.55, 1.42)	1.17 (0.75, 1.83)	0.66
Model 4	ref.	1 (0.62, 1.63)	1 (0.58, 1.74)	0.89 (0.55, 1.46)	1.2 (0.77, 1.87)	0.58
Albumin						
N (%) ^b^	38 (2)	29 (2)	38 (2.6)	38 (2.6)	54 (3.7)	
OR (95% CI)						
Model 1	ref.	0.91 (0.46, 1.8)	1.22 (0.62, 2.39)	1.27 (0.68, 2.37)	2.05 (1.22, 3.42)	0.003
Model 2	ref.	1 (0.47, 2.09)	1.38 (0.58, 3.3)	1.28 (0.58, 2.81)	1.65 (0.87, 3.14)	0.11
Model 3	ref.	1.03 (0.47, 2.24)	1.42 (0.59, 3.42)	1.28 (0.57, 2.86)	1.62 (0.84, 3.12)	0.13
Model 4	ref.	1.03 (0.47, 2.27)	1.41 (0.56, 3.58)	1.33 (0.58, 3.05)	1.56 (0.75, 3.27)	0.16
Bilirubin						
N (%) ^b^	24 (2.2)	27 (2.1)	42 (3.6)	33 (2.6)	43 (3.9)	
OR (95% CI)						
Model 1	ref.	1 (0.52, 1.92)	1.7 (0.82, 3.5)	1.11 (0.53, 2.33)	1.44 (0.73, 2.85)	0.25
Model 2	ref.	0.95 (0.38, 2.38)	1.86 (0.77, 4.45)	1.19 (0.48, 2.94)	1.59 (0.67, 3.75)	0.16
Model 3	ref.	0.95 (0.37, 2.41)	1.85 (0.76, 4.53)	1.18 (0.47, 2.97)	1.58 (0.66, 3.8)	0.16
Model 4	ref.	0.94 (0.36, 2.43)	1.81 (0.72, 4.58)	1.16 (0.44, 3.04)	1.54 (0.63, 3.8)	0.18

^a^ Defined as >47 IU/L in men or >30 IU/L in women for ALT, >33 IU/L for AST, >113 IU/L for ALP, >65 IU/L in men or >36 IU/L in women for GGT, <3.7 g/dL for albumin, and >1.3 mg/dL for bilirubin. ^b^ Percentage weighted using sample weights. Model 1: adjusted for age and gender. Model 2: adjusted for age, gender, race and ethnicity, education, household income, marital status, smoking, alcohol, hepatitis B, hepatitis C and hepatitis E. Model 3: adjusted for age, gender, race and ethnicity, education, household income, marital status, smoking, alcohol, hepatitis B, hepatitis C, hepatitis E and diabetes status. Model 4: adjusted for age, gender, race and ethnicity, education, household income, marital status, smoking, alcohol, hepatitis B, hepatitis C, hepatitis E and BMI. Abbreviations: NHANES, National Health and Nutrition Examination Survey; ALT, alanine aminotransferase; AST, aspartate aminotransferase; ALP, alkaline phosphatase; GGT, gamma-glutamyl transaminase; IQR, interquartile range; CI, confidence interval; OR, odds ratio.

**Table 7:** Associations (OR and 95% CI) between quintiles of interdaily stability and abnormal levels of liver enzymes, albumin and bilirubin, NHANES 2011–2014.

Abnormal Levels of Liver Enzymes ^a^	Interdaily Stability					*p* Trend
	Q1	Q2	Q3	Q4	Q5	
Interdaily stability, median (IQR)	0.4 (0.3, 0.4)	0.5 (0.5, 0.5)	0.6 (0.6, 0.6)	0.7 (0.7, 0.7)	0.8 (0.7, 0.8)	
ALT						
N (%) ^b^	136 (11)	145 (12)	138 (9.9)	114 (9)	131 (11)	
OR (95% CI)						
Model 1	0.93 (0.61, 1.42)	1 (0.63, 1.59)	0.83 (0.55, 1.27)	0.76 (0.53, 1.09)	ref.	0.84
Model 2	0.84 (0.5, 1.4)	1.01 (0.57, 1.78)	0.85 (0.52, 1.38)	0.75 (0.52, 1.1)	ref.	0.91
Model 3	0.79 (0.47, 1.35)	0.96 (0.53, 1.74)	0.81 (0.48, 1.35)	0.73 (0.49, 1.08)	ref.	0.74
Model 4	0.79 (0.46, 1.37)	0.93 (0.51, 1.71)	0.81 (0.48, 1.37)	0.74 (0.5, 1.09)	ref.	0.67
AST						
N (%) ^b^	167 (13)	159 (12)	132 (8.8)	104 (7.9)	109 (9.2)	
OR (95% CI)						
Model 1	1.45 (1.04, 2.01)	1.27 (0.9, 1.79)	0.92 (0.62, 1.36)	0.84 (0.62, 1.12)	ref.	0.01
Model 2	1.32 (0.82, 2.12)	1.26 (0.76, 2.08)	0.95 (0.58, 1.56)	0.79 (0.55, 1.13)	ref.	0.09
Model 3	1.26 (0.77, 2.05)	1.21 (0.73, 2.02)	0.92 (0.55, 1.53)	0.78 (0.53, 1.14)	ref.	0.12
Model 4	1.33 (0.8, 2.21)	1.23 (0.72, 2.1)	0.94 (0.55, 1.6)	0.8 (0.55, 1.17)	ref.	0.11
ALP						
N (%) ^b^	73 (5.1)	33 (2.1)	37 (2.2)	29 (2.5)	34 (2.4)	
OR (95% CI)						
Model 1	3.06 (1.88, 5)	1.11 (0.6, 2.06)	1.13 (0.69, 1.87)	1.14 (0.6, 2.17)	ref.	0.01
Model 2	2.56 (1.26, 5.21)	1.18 (0.54, 2.62)	1.18 (0.6, 2.32)	1.15 (0.47, 2.78)	ref.	0.03
Model 3	2.38 (1.15, 4.95)	1.11 (0.49, 2.49)	1.14 (0.56, 2.33)	1.09 (0.44, 2.71)	ref.	0.04
Model 4	2.59 (1.19, 5.66)	1.16 (0.5, 2.65)	1.08 (0.47, 2.52)	1.06 (0.42, 2.67)	ref.	0.04
GGT						
N (%) ^b^	159 (12)	137 (9.9)	132 (8.9)	111 (8)	116 (8.5)	
OR (95% CI)						
Model 1	1.87 (1.29, 2.71)	1.42 (0.95, 2.13)	1.21 (0.83, 1.75)	0.99 (0.69, 1.42)	ref.	0.001
Model 2	1.72 (1.05, 2.81)	1.49 (0.93, 2.39)	1.11 (0.74, 1.69)	0.99 (0.64, 1.53)	ref.	0.02
Model 3	1.63 (1, 2.65)	1.42 (0.86, 2.35)	1.07 (0.7, 1.63)	0.96 (0.61, 1.51)	ref.	0.02
Model 4	1.6 (0.93, 2.77)	1.35 (0.79, 2.29)	1.07 (0.68, 1.67)	0.98 (0.62, 1.55)	ref.	0.04
Albumin						
N (%) ^b^	69 (4.9)	45 (2)	36 (2.9)	29 (2)	21 (1.2)	
OR (95% CI)						
Model 1	5.65 (2.85, 11.19)	2.12 (0.93, 4.83)	2.9 (1.34, 6.28)	1.78 (0.72, 4.36)	ref.	<0.001
Model 2	5.09 (1.94, 13.39)	1.96 (0.66, 5.81)	2.98 (1.04, 8.58)	1.8 (0.61, 5.35)	ref.	0.001
Model 3	4.7 (1.7, 13.03)	1.82 (0.58, 5.76)	2.81 (0.93, 8.45)	1.7 (0.54, 5.35)	ref.	0.002
Model 4	4.43 (1.56, 12.58)	1.68 (0.53, 5.29)	2.52 (0.8, 7.89)	1.71 (0.54, 5.43)	ref.	0.004
Bilirubin						
N (%) ^b^	47 (3.5)	39 (4.4)	33 (2.9)	30 (2.1)	23 (1.5)	
OR (95% CI)						
Model 1	1.72 (0.89, 3.33)	2.37 (1, 5.62)	1.63 (0.89, 2.99)	1.3 (0.61, 2.76)	ref.	0.02
Model 2	1.43 (0.66, 3.12)	2.3 (0.88, 6.01)	1.55 (0.77, 3.13)	1.16 (0.49, 2.74)	ref.	0.06
Model 3	1.42 (0.64, 3.15)	2.29 (0.86, 6.1)	1.54 (0.76, 3.15)	1.16 (0.48, 2.77)	ref.	0.07
Model 4	1.4 (0.63, 3.13)	2.36 (0.85, 6.58)	1.55 (0.73, 3.27)	1.14 (0.46, 2.83)	ref.	0.07

^a^ Defined as >47 IU/L in men or >30 IU/L in women for ALT, >33 IU/L for AST, >113 IU/L for ALP, >65 IU/L in men or >36 IU/L in women for GGT, <3.7 g/dL for albumin, and >1.3 mg/dL for bilirubin. ^b^ Percentage weighted using sample weights. Model 1: adjusted for age and gender. Model 2: adjusted for age, gender, race and ethnicity, education, household income, marital status, smoking, alcohol, hepatitis B, hepatitis C and hepatitis E. Model 3: adjusted for age, gender, race and ethnicity, education, household income, marital status, smoking, alcohol, hepatitis B, hepatitis C, hepatitis E and diabetes status. Model 4: adjusted for age, gender, race and ethnicity, education, household income, marital status, smoking, alcohol, hepatitis B, hepatitis C, hepatitis E and BMI. Abbreviations: NHANES, National Health and Nutrition Examination Survey; ALT, alanine aminotransferase; AST, aspartate aminotransferase; ALP, alkaline phosphatase; GGT, gamma-glutamyl transaminase; IQR, interquartile range; CI, confidence interval; OR, odds ratio.

**Table 8:** Associations (OR and 95% CI) between quintiles of intradaily variability and abnormal levels of liver enzymes, albumin and bilirubin, NHANES 2011–2014.

Abnormal Levels of Liver Enzymes ^a^	Intradaily Variability					*p* Trend
	Q1	Q2	Q3	Q4	Q5	
Intradaily variability, median (IQR)	0.4 (0.4, 0.5)	0.6 (0.5, 0.6)	0.7 (0.6, 0.7)	0.8 (0.8, 0.8)	1.0 (0.9, 1.1)	
ALT						
N (%) ^b^	135 (11)	123 (9.3)	141 (11)	152 (12)	113 (9)	
OR (95% CI)						
Model 1	ref.	0.79 (0.52, 1.18)	0.97 (0.64, 1.49)	1.06 (0.7, 1.62)	0.81 (0.58, 1.14)	0.81
Model 2	ref.	0.99 (0.62, 1.59)	1.15 (0.71, 1.87)	1.35 (0.83, 2.2)	1.01 (0.69, 1.47)	0.35
Model 3	ref.	0.98 (0.6, 1.59)	1.1 (0.67, 1.83)	1.29 (0.78, 2.14)	0.93 (0.63, 1.36)	0.67
Model 4	ref.	1.01 (0.61, 1.67)	1.15 (0.69, 1.92)	1.31 (0.79, 2.18)	0.97 (0.65, 1.44)	0.53
AST						
N (%) ^b^	153 (12)	112 (8.3)	124 (9.7)	135 (9.2)	147 (11)	
OR (95% CI)						
Model 1	ref.	0.7 (0.47, 1.04)	0.86 (0.57, 1.29)	0.79 (0.54, 1.17)	0.97 (0.68, 1.4)	0.94
Model 2	ref.	0.87 (0.53, 1.44)	1.04 (0.62, 1.72)	0.99 (0.58, 1.7)	1.32 (0.75, 2.34)	0.28
Model 3	ref.	0.87 (0.52, 1.45)	1 (0.59, 1.68)	0.95 (0.53, 1.69)	1.22 (0.68, 2.17)	0.44
Model 4	ref.	0.87 (0.51, 1.48)	1.03 (0.6, 1.77)	0.98 (0.55, 1.74)	1.3 (0.71, 2.38)	0.32
ALP						
N (%) ^b^	38 (2.8)	30 (2)	34 (2.4)	33 (1.6)	71 (5.2)	
OR (95% CI)						
Model 1	ref.	0.63 (0.34, 1.15)	0.71 (0.35, 1.47)	0.48 (0.25, 0.92)	1.61 (0.97, 2.68)	0.15
Model 2	ref.	0.81 (0.37, 1.8)	1.21 (0.55, 2.69)	0.69 (0.3, 1.59)	2.38 (1.2, 4.71)	0.03
Model 3	ref.	0.79 (0.34, 1.85)	1.14 (0.49, 2.65)	0.64 (0.27, 1.52)	2.09 (1.02, 4.27)	0.06
Model 4	ref.	0.8 (0.35, 1.82)	1.06 (0.45, 2.46)	0.68 (0.28, 1.66)	2.44 (1.16, 5.13)	0.04
GGT						
N (%) ^b^	120 (9.3)	122 (8.3)	134 (8.9)	141 (10)	138 (10)	
OR (95% CI)						
Model 1	ref.	0.8 (0.61, 1.06)	0.83 (0.59, 1.15)	0.98 (0.77, 1.26)	1.01 (0.73, 1.41)	0.52
Model 2	ref.	1.02 (0.73, 1.42)	1.06 (0.73, 1.56)	1.36 (0.99, 1.87)	1.42 (0.96, 2.1)	0.04
Model 3	ref.	1 (0.71, 1.41)	1.01 (0.68, 1.51)	1.28 (0.92, 1.77)	1.29 (0.89, 1.88)	0.09
Model 4	ref.	1.04 (0.73, 1.48)	1.06 (0.71, 1.58)	1.32 (0.93, 1.87)	1.36 (0.89, 2.09)	0.08
Albumin						
N (%) ^b^	20 (1.5)	30 (1.8)	28 (1.7)	46 (3.5)	76 (4.3)	
OR (95% CI)						
Model 1	ref.	1.07 (0.46, 2.51)	0.94 (0.37, 2.38)	2.07 (0.87, 4.9)	2.72 (1.23, 6.03)	0.001
Model 2	ref.	1.54 (0.55, 4.37)	1.36 (0.44, 4.21)	3.2 (1.01, 10.07)	3.34 (1.05, 10.68)	0.01
Model 3	ref.	1.51 (0.52, 4.39)	1.27 (0.4, 4.06)	3.01 (0.91, 10.02)	2.97 (0.91, 9.72)	0.01
Model 4	ref.	1.54 (0.5, 4.74)	1.16 (0.33, 4.08)	2.82 (0.83, 9.57)	3 (0.86, 10.49)	0.02
Bilirubin						
N (%) ^b^	25 (1.7)	32 (2.5)	32 (2.7)	36 (4.4)	47 (3)	
OR (95% CI)						
Model 1	ref.	1.78 (0.75, 4.22)	2.11 (1.05, 4.23)	3.49 (1.49, 8.14)	2.21 (0.98, 5)	0.003
Model 2	ref.	2.1 (0.62, 7.16)	2.21 (0.78, 6.28)	4.05 (1.13, 14.51)	2.12 (0.59, 7.59)	0.05
Model 3	ref.	2.1 (0.6, 7.37)	2.21 (0.76, 6.41)	4.03 (1.08, 15.01)	2.11 (0.57, 7.85)	0.06
Model 4	ref.	2.11 (0.55, 8.1)	2.31 (0.74, 7.2)	4.15 (1.04, 16.46)	2.16 (0.54, 8.69)	0.06

^a^ Defined as >47 IU/L in men or >30 IU/L in women for ALT, >33 IU/L for AST, >113 IU/L for ALP, >65 IU/L in men or >36 IU/L in women for GGT, <3.7 g/dL for albumin, and >1.3 mg/dL for bilirubin. ^b^ Percentage weighted using sample weights. Model 1: adjusted for age and gender. Model 2: adjusted for age, gender, race and ethnicity, education, household income, marital status, smoking, alcohol, hepatitis B, hepatitis C and hepatitis E. Model 3: adjusted for age, gender, race and ethnicity, education, household income, marital status, smoking, alcohol, hepatitis B, hepatitis C, hepatitis E and diabetes status. Model 4: adjusted for age, gender, race and ethnicity, education, household income, marital status, smoking, alcohol, hepatitis B, hepatitis C, hepatitis E and BMI. Abbreviations: NHANES, National Health and Nutrition Examination Survey; ALT, alanine aminotransferase; AST, aspartate aminotransferase; ALP, alkaline phosphatase; GGT, gamma-glutamyl transaminase; IQR, interquartile range; CI, confidence interval; OR, odds ratio.

## Data Availability

NHANES datasets and documents are available at https://wwwn.cdc.gov/nchs/nhanes/ (accessed on 12 June 2023).

## Supplementary Materials

The following supporting information can be downloaded at: https://www.mdpi.com/article/10.3390/clockssleep5040045/s1, Table S1: Pearson correlation coefficients between rest–activity rhythm parameters, NHANES 2011–2014; Table S2. Associations (Beta and 95% CI) between quintiles of rest–activity rhythm characteristics and level of ALT, AST, ALP, GGT, albumin and bilirubin, NHANES 2011–2014; Table S3. Associations (Beta and 95% CI) between quintiles of rest–activity rhythm characteristics and level of ALT, AST, ALP, GGT, albumin and bilirubin, model 3 and 4, NHANES 2011–2014; Table S4. Associations (OR and 95% CI) between quintiles of rest–activity rhythm characteristics and abnormal levels of ALT, AST, ALP, GGT, albumin and bilirubin, adjusting for physical activity and sleeping duration, NHANES 2011–2014; Table S5. Associations (OR and 95% CI) between quintiles of F statistic and abnormal levels of ALT, AST, ALP, GGT, albumin and bilirubin, after removing night shift worker proxied by L5MD, NHANES 2011–2014; Table S6. Associations (OR and 95% CI) between quintiles of rest–activity rhythm characteristics and abnormal levels of ALT, AST, ALP and GGT, stratified by alcohol drinking status, NHANES 2011–2014; Table S7. Associations (OR and 95% CI) between quintiles of rest–activity rhythm characteristics and abnormal levels of ALT, AST, ALP and GGT, stratified by BMI, NHANES 2011–2014; Table S8. Associations (OR and 95% CI) between rest–activity rhythm characteristics quintiles and abnormal levels of ALT, AST, ALP and GGT, stratified by diabetes status, NHANES 2011–2014; Table S9. Associations (OR and 95% CI) between rest–activity rhythm characteristics quintiles and abnormal levels of ALT, AST, ALP, and GGT, stratified by sessions attended, NHANES 2011–2014; Table S10. Associations (OR and 95% CI) between impaired rhythmicity score and abnormal levels of liver enzymes, albumin, and bilirubin, stratified by diabetes status, NHANES 2011–2014; Table S11. Associations (OR and 95% CI) between rest–activity rhythm characteristics quintiles and abnormal liver enzyme score, NHANES 2011–2014.
