# Supplementary material for: Associations between Rest–Activity Rhythms and Liver Function Tests: The US National Health and Nutrition Examination Survey, 2011–2014
Source: Clocks Sleep. 2023 Nov 2;5(4):667–85. doi: 10.3390/clockssleep5040045 (PMC10660688; doi:10.3390/clockssleep5040045)
Supplement: Supplementary file 1 [file clockssleep-05-00045-s001.zip › clockssleep-2651693-supplementary.pdf]

Table S1. Pearson correlation coefficients between rest-activity rhythm parameters, NHANES 2011 – 2014

	F Statistic	Amplitude	Mesor	Amplitude:Mesor	Acrophase	Interdaily stability	Intradaily variability
**F Statistic**	1	0.14	0.17	0.23	-0.15	0.72	-0.45
**Amplitude**		1	0.48	0.75	0.00	0.10	-0.23
**Mesor**			1	0.04	0.00	0.18	-0.29
**Amplitude:Mesor**				1	-0.01	0.17	-0.32
**Acrophase**					1	-0.21	0.01
**Interdaily stability**						1	-0.43
**Intradaily variability**							1

Table S2. Associations (Beta and 95% CI) between quintiles of rest-activity rhythm characteristics and level of ALT, AST, ALP, GGT, albumin and bilirubin, NHANES 2011 – 2014

	ALT, log(U/L)		AST, log(U/L)		ALP, log(U/L)		GGT, log(U/L)		Albumin, g/dL		Bilirubin, log(mg/dL)	
	median (IQR)	Beta (95% CI)	median (IQR)	Beta (95% CI)	median (IQR)	Beta (95% CI)	median (IQR)	Beta (95% CI)	median (IQR)	Beta (95% CI)	median (IQR)	Beta (95% CI)
**F Statistic**												
Q1	3.0 (2.8, 3.4)	0.06 (-0.002, 0.11)	3.1 (2.9, 3.3)	0.05 (-0.01, 0.10)	4.2 (4.0, 4.4)	0.08 (0.04, 0.12)	3.0 (2.7, 3.5)	0.14 (0.06, 0.23)	4.2 (4.0-4.5)	-0.11 (-0.15, -0.06)	-0.5 (-0.7, -0.2)	-0.03 (-0.08, 0.02)
Q2	3.0 (2.8, 3.4)	0.09 (0.03, 0.14)	3.1 (2.9, 3.3)	0.04 (-0.004, 0.08)	4.2 (4.0, 4.4)	0.04 (0.004, 0.09)	3.0 (2.6, 3.4)	0.14 (0.07, 0.22)	4.3 (4.1-4.5)	-0.05 (-0.09, -0.01)	-0.5 (-0.7, -0.2)	-0.05 (-0.12, 0.01)
Q3	3.0 (2.8, 3.3)	0.02 (-0.02, 0.07)	3.1 (2.9, 3.3)	0.01 (-0.02, 0.05)	4.2 (4.0, 4.3)	0.03 (-0.01, 0.06)	2.9 (2.6, 3.4)	0.07 (0.01, 0.13)	4.3 (4.0-4.5)	-0.06 (-0.11, -0.02)	-0.5 (-0.7, -0.2)	-0.002 (-0.05, 0.05)
Q4	3.0 (2.8, 3.3)	0.01 (-0.04, 0.06)	3.1 (2.9, 3.3)	-0.002 (-0.04, 0.03)	4.1 (3.9, 4.3)	-0.01 (-0.04, 0.03)	2.9 (2.6, 3.3)	0.03 (-0.03, 0.10)	4.3 (4.1-4.5)	-0.02 (-0.06, 0.02)	-0.5 (-0.7, -0.2)	0.0004 (-0.03, 0.03)
Q5	3.0 (2.8, 3.3)	ref.	3.1 (2.9, 3.3)	ref.	4.1 (3.9, 4.3)	ref.	2.9 (2.6, 3.3)	ref.	4.3 (4.1-4.5)	ref.	-0.5 (-0.7, -0.2)	ref.
P trend		0.004		0.02		<0.001		<0.001		<0.001		0.04
**Amplitude**												
Q1	3.0 (2.8, 3.3)	0.04 (-0.01, 0.09)	3.1 (2.9, 3.3)	0.04 (-0.01, 0.08)	4.2 (4.0, 4.4)	0.04 (0, 0.09)	3.0 (2.7, 3.5)	0.12 (0.02, 0.21)	4.2 (4.0- 4.4)	-0.09 (-0.14, -0.04)	-0.5 (-0.7, -0.2)	-0.04 (-0.09, 0.01)
Q2	3.0 (2.8, 3.3)	0.05 (0.005, 0.09)	3.1 (2.9, 3.3)	0.02 (-0.02, 0.06)	4.2 (4.0, 4.3)	0.02 (-0.02, 0.05)	3.0 (2.6, 3.4)	0.08 (0.01, 0.15)	4.3 (4.0-4.5)	-0.04 (-0.09, 0.001)	-0.5 (-0.7, -0.2)	-0.03 (-0.09, 0.03)
Q3	3.0 (2.8, 3.3)	0.03 (-0.02, 0.08)	3.1 (2.9, 3.3)	0.01 (-0.02, 0.05)	4.1 (4.0, 4.3)	0.0001 (-0.04, 0.04)	2.9 (2.6, 3.3)	0.03 (-0.06, 0.12)	4.3 (4.1-4.5)	-0.02 (-0.05, 0.01)	-0.5 (-0.7, -0.2)	-0.01 (-0.05, 0.03)
Q4	3.0 (2.8, 3.3)	0.01 (-0.04, 0.06)	3.1 (2.9, 3.3)	-0.004 (-0.03, 0.03)	4.1 (4.0, 4.3)	0.005 (-0.04, 0.05)	2.9 (2.6, 3.3)	0.02 (-0.06, 0.09)	4.3 (4.1-4.5)	-0.01 (-0.05, 0.02)	-0.5 (-0.7, -0.2)	0.01 (-0.04, 0.06)
Q5	3.0 (2.8, 3.3)	ref.	3.1 (2.9, 3.3)	ref.	4.1 (4.0, 4.3)	ref.	2.9 (2.6, 3.4)	ref.	4.3 (4.1-4.5)	ref.	-0.5 (-0.7, -0.2)	ref.
P trend		0.04		0.09		0.03		0.004		0.002		0.06
**Mesor**												
Q1	3.0 (2.8, 3.4)	0.04 (-0.02, 0.1)	3.1 (2.9, 3.3)	0.02 (-0.02, 0.07)	4.2 (4.0, 4.4)	0.02 (-0.02, 0.06)	3.0 (2.7, 3.5)	0.08 (-0.01, 0.17)	4.2 (4.0, 4.5)	-0.04 (-0.09, 0.01)	-0.5 (-0.7, -0.2)	-0.01 (-0.07, 0.05)
Q2	3.0 (2.8, 3.3)	0.01 (-0.04, 0.06)	3.1 (2.9, 3.3)	-0.001 (-0.03, 0.03)	4.2 (4.0, 4.4)	-0.01 (-0.06, 0.04)	2.9 (2.6, 3.4)	0.004 (-0.07, 0.08)	4.3 (4.1, 4.5)	-0.01 (-0.04, 0.02)	-0.5 (-0.7, -0.2)	0.02 (-0.04, 0.08)
Q3	3.0 (2.8, 3.3)	0.02 (-0.03, 0.08)	3.1 (2.9, 3.3)	0.02 (-0.02, 0.06)	4.2 (4.0, 4.3)	-0.01 (-0.06, 0.03)	3.0 (2.6, 3.4)	0.06 (-0.02, 0.13)	4.3 (4.1, 4.5)	0.001 (-0.04, 0.04)	-0.5 (-0.7, -0.2)	0.02 (-0.04, 0.08)
Q4	3.0 (2.8, 3.3)	0.01 (-0.04, 0.07)	3.1 (2.9, 3.3)	0.03 (-0.02, 0.07)	4.2 (4.0, 4.3)	-0.02 (-0.06, 0.02)	2.9 (2.6, 3.3)	-0.01 (-0.09, 0.07)	4.3 (4.1, 4.5)	0.01 (-0.02, 0.05)	-0.5 (-0.7, -0.2)	0.06 (0.01, 0.12)
Q5	3.0 (2.8, 3.3)	ref.	3.1 (2.9, 3.3)	ref.	4.2 (4.0, 4.3)	ref.	2.9 (2.6, 3.3)	ref.	4.3 (4.1, 4.5)	ref.	-0.5 (-0.7, -0.2)	ref.
P trend		0.25		0.74		0.22		0.049		0.04		0.21
**Amplitude:Mesor**												
Q1	3.0 (2.8, 3.4)	0.04 (-0.01, 0.09)	3.1 (2.9, 3.3)	0.06 (0.01, 0.10)	4.2 (4.0, 4.4)	0.04 (-0.003, 0.08)	3.0 (2.7, 3.5)	0.10 (0.01, 0.20)	4.2 (4.0, 4.4)	-0.09 (-0.13, -0.04)	-0.5 (-0.7, -0.2)	-0.02 (-0.06, 0.03)
Q2	3.0 (2.8, 3.3)	-0.01 (-0.06, 0.04)	3.1 (2.9, 3.3)	-0.003 (-0.04, 0.03)	4.2 (4.0, 4.3)	0.005 (-0.03, 0.04)	2.9 (2.6, 3.3)	-0.01 (-0.08, 0.07)	4.3 (4.1, 4.5)	-0.002 (-0.04, 0.04)	-0.5 (-0.7, -0.2)	-0.01 (-0.06, 0.04)
Q3	3.0 (2.8, 3.3)	-0.01 (-0.06, 0.04)	3.1 (2.9, 3.3)	-0.01 (-0.05, 0.02)	4.1 (4.0, 4.3)	-0.005 (-0.04, 0.03)	2.9 (2.6, 3.3)	-0.01 (-0.09, 0.08)	4.3 (4.1, 4.5)	-0.01 (-0.04, 0.02)	-0.5 (-0.7, -0.2)	-0.01 (-0.05, 0.03)
Q4	3.0 (2.8, 3.3)	-0.02 (-0.07, 0.02)	3.1 (2.9, 3.3)	-0.004 (-0.04, 0.03)	4.1 (4.0, 4.3)	-0.01 (-0.04, 0.03)	2.9 (2.6, 3.4)	-0.02 (-0.11, 0.06)	4.3 (4.1, 4.5)	0.004 (-0.03, 0.03)	-0.5 (-0.7, -0.2)	0.03 (-0.01, 0.07)
Q5	3.0 (2.8, 3.3)	ref.	3.1 (2.9, 3.3)	ref.	4.1 (4.0, 4.3)	ref.	2.9 (2.6, 3.4)	ref.	4.3 (4.1, 4.5)	ref.	-0.5 (-0.7, -0.2)	ref.
P trend		0.09		0.046		0.04		0.04		0.003		0.09
**Acrophase**												
Q1	3.0 (2.8, 3.3)	ref.	3.1 (2.9, 3.3)	ref.	4.2 (4.0, 4.3)	ref.	2.9 (2.6, 3.3)	ref.	4.3 (4.1, 4.5)	ref.	-0.5 (-0.7, -0.2)	ref.
Q2	3.0 (2.8, 3.3)	-0.01 (-0.05, 0.04)	3.1 (2.9, 3.3)	-0.005 (-0.05, 0.04)	4.2 (4.0, 4.3)	-0.02 (-0.05, 0.02)	2.9 (2.6, 3.4)	-0.02 (-0.09, 0.06)	4.3 (4.1, 4.5)	-0.01 (-0.05, 0.03)	-0.5 (-0.7, -0.2)	-0.03 (-0.08, 0.03)
Q3	3.0 (2.8, 3.3)	-0.02 (-0.06, 0.03)	3.1 (2.9, 3.3)	-0.01 (-0.04, 0.01)	4.2 (4.0, 4.3)	-0.02 (-0.06, 0.03)	2.9 (2.6, 3.4)	-0.02 (-0.09, 0.06)	4.3 (4.1, 4.5)	0.001 (-0.03, 0.03)	-0.5 (-0.7, -0.2)	-0.01 (-0.06, 0.04)
Q4	3.0 (2.8, 3.4)	-0.04 (-0.09, 0.01)	3.1 (2.9, 3.3)	-0.03 (-0.06, 0.01)	4.2 (4.0, 4.4)	-0.03 (-0.06, 0.01)	2.9 (2.6, 3.4)	-0.04 (-0.12, 0.04)	4.3 (4.0, 4.5)	-0.01 (-0.05, 0.03)	-0.5 (-0.7, -0.2)	-0.03 (-0.09, 0.02)
Q5	3.0 (2.8, 3.4)	0.03 (-0.03, 0.08)	3.1 (3.0, 3.3)	0.01 (-0.03, 0.04)	4.2 (4.0, 4.4)	0.02 (-0.01, 0.06)	3.0 (2.7, 3.4)	0.04 (-0.04, 0.13)	4.3 (4.1, 4.5)	-0.03 (-0.07, 0.02)	-0.5 (-0.7, -0.2)	-0.01 (-0.07, 0.04)
P trend		0.84		0.62		0.50		0.54		0.08		0.78
**Interdaily stability**												
Q1	3.1 (2.8, 3.4)	0.02 (-0.04, 0.07)	3.1 (2.9, 3.3)	0.03 (-0.02, 0.07)	4.2 (4.0, 4.4)	0.06 (0.02, 0.1)	2.9 (2.6, 3.4)	0.05 (-0.04, 0.14)	4.3 (4.0, 4.5)	-0.09 (-0.13, -0.05)	-0.5 (-0.7, -0.2)	-0.03 (-0.1, 0.03)
Q2	3.0 (2.8, 3.4)	0.01 (-0.05, 0.08)	3.1 (2.9, 3.3)	0.02 (-0.03, 0.07)	4.1 (3.9, 4.3)	0.03 (0, 0.07)	2.9 (2.6, 3.4)	0.06 (-0.01, 0.14)	4.3 (4.1, 4.5)	-0.05 (-0.09, 0)	-0.5 (-0.7, -0.2)	-0.01 (-0.06, 0.03)
Q3	3.0 (2.8, 3.3)	-0.01 (-0.05, 0.04)	3.1 (2.9, 3.3)	-0.01 (-0.04, 0.03)	4.1 (3.9, 4.3)	0.01 (-0.03, 0.05)	3.0 (2.6, 3.4)	0.03 (-0.04, 0.09)	4.3 (4.1, 4.5)	-0.07 (-0.12, -0.03)	-0.5 (-0.7, -0.2)	-0.01 (-0.05, 0.03)
Q4	3.0 (2.8, 3.3)	-0.03 (-0.08, 0.02)	3.1 (2.9, 3.3)	-0.02 (-0.05, 0.02)	4.1 (4.0, 4.3)	0.02 (-0.03, 0.06)	2.9 (2.6, 3.3)	-0.02 (-0.09, 0.04)	4.3 (4.1, 4.5)	-0.02 (-0.05, 0.02)	-0.5 (-0.7, -0.2)	-0.03 (-0.07, 0.02)
Q5	3.0 (2.8, 3.3)	ref.	3.1 (3.0, 3.3)	ref.	4.1 (4.0, 4.3)	ref.	2.9 (2.6, 3.3)	ref.	4.3 (4.1, 4.5)	ref.	-0.5 (-0.7, -0.2)	ref.
P trend		0.23		0.07		0.001		0.048		<0.001		0.36
**Intradaily variability**												
Q1	3.0 (2.8, 3.4)	ref.	3.1 (3.0, 3.3)	ref.	4.2 (4.0, 4.4)	ref.	2.9 (2.6, 3.4)	ref.	4.3 (4.1, 4.5)	ref.	-0.5 (-0.7, -0.2)	ref.
Q2	3.0 (2.8, 3.3)	0.01 (-0.05, 0.06)	3.1 (2.9, 3.3)	-0.01 (-0.05, 0.02)	4.1 (4.0, 4.3)	0 (-0.04, 0.04)	2.9 (2.6, 3.3)	-0.01 (-0.08, 0.06)	4.3 (4.1, 4.5)	-0.01 (-0.04, 0.03)	-0.5 (-0.7, -0.2)	0.02 (-0.03, 0.07)
Q3	3.0 (2.8, 3.3)	0.02 (-0.03, 0.07)	3.1 (2.9, 3.3)	0.01 (-0.04, 0.05)	4.2 (4.0, 4.3)	0 (-0.04, 0.05)	2.9 (2.6, 3.3)	-0.01 (-0.08, 0.07)	4.3 (4.1, 4.5)	-0.01 (-0.04, 0.02)	-0.5 (-0.7, -0.2)	0.02 (-0.03, 0.08)
Q4	3.0 (2.8, 3.4)	0.04 (-0.01, 0.1)	3.1 (3.0, 3.3)	0.03 (-0.01, 0.07)	4.1 (3.9, 4.3)	-0.02 (-0.06, 0.03)	2.9 (2.6, 3.4)	0.05 (-0.01, 0.11)	4.3 (4.1, 4.5)	-0.04 (-0.09, 0)	-0.5 (-0.7, -0.2)	0.02 (-0.05, 0.09)
Q5	3.0 (2.8, 3.3)	0.03 (-0.03, 0.08)	3.1 (2.9, 3.3)	0.03 (-0.02, 0.08)	4.1 (3.9, 4.3)	0.01 (-0.02, 0.05)	2.9 (2.6, 3.3)	0.04 (-0.03, 0.11)	4.3 (4.1, 4.5)	-0.04 (-0.07, 0)	-0.4 (-0.7, -0.2)	0.02 (-0.04, 0.07)
P trend		0.09		0.045		0.65		0.07		0.01		0.59

Regression results presented as log-transformed liver enzyme levels.
Results based on model 2 adjusted for age, gender, race and ethnicity, education, household income, marital status, smoking, alcohol, hepatitis B, hepatitis C and hepatitis E.
Abbreviations: NHANES, National Health and Nutrition Examination Survey; ALT, alanine aminotransferase; AST, aspartate aminotransferase; ALP, alkaline phosphatase; GGT, gamma glutamyl transaminase; CI, confidence interval

Table S3. Associations (Beta and 95% CI) between quintiles of rest-activity rhythm characteristics and level of ALT, AST, ALP, GGT, albumin and bilirubin, model 3 and 4, NHANES 2011 – 2014

	ALT, log(U/L)		AST, log(U/L)		ALP, log(U/L)		GGT, log(U/L)		Albumin, g/dL		Bilirubin, log(mg/dL)	
	Model 3	Model 4	Model 3	Model 4	Model 3	Model 4	Model 3	Model 4	Model 3	Model 4	Model 3	Model 4
**F Statistic**												
Q1	0.04 (-0.02, 0.1)	0.02 (-0.04, 0.08)	0.04 (-0.01, 0.1)	0.04 (-0.02, 0.1)	0.08 (0.04, 0.11)	0.07 (0.03, 0.11)	0.12 (0.04, 0.2)	0.09 (-0.003, 0.18)	-0.1 (-0.15, -0.05)	-0.08 (-0.12, -0.03)	-0.03 (-0.08, 0.03)	-0.01 (-0.07, 0.05)
Q2	0.08 (0.02, 0.14)	0.06 (0.003, 0.12)	0.03 (-0.01, 0.08)	0.03 (-0.01, 0.08)	0.04 (-0.003, 0.08)	0.03 (-0.01, 0.08)	0.12 (0.04, 0.21)	0.1 (0.02, 0.18)	-0.04 (-0.09, 0.0002)	-0.03 (-0.07, 0.01)	-0.05 (-0.11, 0.01)	-0.04 (-0.1, 0.03)
Q3	0.02 (-0.03, 0.06)	-0.002 (-0.05, 0.05)	0.01 (-0.03, 0.05)	0.01 (-0.03, 0.05)	0.03 (-0.01, 0.06)	0.02 (-0.02, 0.06)	0.06 (-0.003, 0.12)	0.03 (-0.03, 0.1)	-0.06 (-0.1, -0.01)	-0.04 (-0.09, 0.003)	0.001 (-0.05, 0.06)	0.01 (-0.05, 0.07)
Q4	0.01 (-0.04, 0.05)	-0.01 (-0.05, 0.04)	-0003 (-0.04, 0.03)	-0.004 (-0.04, 0.03)	-0.01 (-0.05, 0.03)	-0.01 (-0.05, 0.03)	0.03 (-0.04, 0.09)	0.01 (-0.05, 0.07)	-0.02 (-0.06, 0.02)	-0.01 (-0.04, 0.02)	0.001 (-0.03, 0.03)	0.01 (-0.03, 0.04)
Q5	ref.	ref.	ref.	ref.	ref.	ref.	ref.	ref.	ref.	ref.	ref.	ref.
P trend	0.01	0.07	0.03	0.04	<0.001	<0.001	0.002	0.01	0.001	0.003	0.14	0.35
**Amplitude**												
Q1	0.02 (-0.03, 0.07)	0.01 (-0.04, 0.06)	0.03 (-0.02, 0.08)	0.03 (-0.02, 0.09)	0.03 (-0.01, 0.08)	0.03 (-0.02, 0.08)	0.09 (-0.01, 0.18)	0.07 (-0.02, 0.17)	-0.08 (-0.13, -0.03)	-0.07 (-0.12, -0.02)	-0.04 (-0.09, 0.02)	-0.03 (-0.08, 0.03)
Q2	0.04 (-0.01, 0.09)	0.03 (-0.02, 0.08)	0.02 (-0.02, 0.06)	0.02 (-0.02, 0.06)	0.01 (-0.02, 0.04)	0.01 (-0.03, 0.04)	0.06 (-0.02, 0.13)	0.05 (-0.03, 0.12)	-0.04 (-0.08, 0.01)	-0.03 (-0.07, 0.02)	-0.03 (-0.09, 0.03)	-0.02 (-0.08, 0.04)
Q3	0.02 (-0.02, 0.07)	0.02 (-0.02, 0.07)	0.01 (-0.02, 0.05)	0.01 (-0.02, 0.05)	-0.002 (-0.04, 0.04)	-0.003 (-0.05, 0.04)	0.02 (-0.07, 0.12)	0.02 (-0.07, 0.11)	-0.01 (-0.05, 0.02)	-0.01 (-0.04, 0.02)	-0.01 (-0.05, 0.03)	-0.01 (-0.05, 0.03)
Q4	0.01 (-0.04, 0.06)	0.01 (-0.04, 0.05)	-0.005 (-0.04, 0.03)	-0.004 (-0.04, 0.03)	0.004 (-0.04, 0.05)	0.003 (-0.04, 0.05)	0.01 (-0.06, 0.09)	0.01 (-0.07, 0.09)	-0.01 (-0.05, 0.02)	-0.01 (-0.05, 0.03)	0.01 (-0.04, 0.06)	0.01 (-0.04, 0.07)
Q5	ref.	ref.	ref.	ref.	ref.	ref.	ref.	ref.	ref.	ref.	ref.	ref.
P trend	0.13	0.31	0.15	0.14	0.07	0.14	0.03	0.06	0.005	0.01	0.08	0.16
**Mesor**												
Q1	0.03 (-0.03, 0.09)	0.01 (-0.05, 0.08)	0.02 (-0.03, 0.07)	0.02 (-0.03, 0.07)	0.01 (-0.03, 0.06)	0.01 (-0.04, 0.05)	0.06 (-0.03, 0.14)	0.04 (-0.06, 0.13)	-0.03 (-0.08, 0.02)	-0.02 (-0.06, 0.03)	-0.01 (-0.07, 0.06)	0.01 (-0.06, 0.07)
Q2	-0.0002 (-0.05, 0.05)	-0.01 (-0.06, 0.04)	-0.003 (-0.03, 0.03)	-0.003 (-0.03, 0.03)	-0.01 (-0.06, 0.03)	-0.02 (-0.07, 0.03)	-0.01 (-0.08, 0.07)	-0.01 (-0.09, 0.06)	-0.005 (-0.04, 0.03)	0.001 (-0.03, 0.04)	0.02 (-0.04, 0.08)	0.03 (-0.03, 0.09)
Q3	0.02 (-0.03, 0.07)	0.01 (-0.04, 0.07)	0.02 (-0.02, 0.06)	0.02 (-0.02, 0.06)	-0.02 (-0.06, 0.03)	-0.02 (-0.06, 0.03)	0.05 (-0.03, 0.13)	0.04 (-0.04, 0.11)	0.004 (-0.04, 0.05)	0.01 (-0.03, 0.05)	0.02 (-0.04, 0.08)	0.03 (-0.03, 0.09)
Q4	0.01 (-0.04, 0.07)	0.02 (-0.04, 0.07)	0.03 (-0.02, 0.07)	0.03 (-0.02, 0.08)	-0.02 (-0.07, 0.02)	-0.02 (-0.07, 0.03)	-0.02 (-0.1, 0.07)	-0.01 (-0.09, 0.08)	0.01 (-0.02, 0.05)	0.01 (-0.03, 0.05)	0.07 (0.01, 0.12)	0.06 (0.01, 0.12)
Q5	ref.	ref.	ref.	ref.	ref.	ref.	ref.	ref.	ref.	ref.	ref.	ref.
P trend	0.46	0.99	0.95	0.98	0.35	0.60	0.14	0.44	0.05	0.21	0.24	0.54
**Amplitude:Mesor**												
Q1	0.03 (-0.02, 0.07)	0.03 (-0.03, 0.08)	0.05 (-0.001, 0.1)	0.06 (0.001, 0.11)	0.03 (-0.01, 0.08)	0.04 (-0.01, 0.08)	0.08 (-0.02, 0.17)	0.08 (-0.02, 0.18)	-0.08 (-0.12, -0.03)	-0.07 (-0.12, -0.02)	-0.01 (-0.06, 0.03)	-0.01 (-0.06, 0.04)
Q2	-0.02 (-0.07, 0.03)	-0.01 (-0.06, 0.04)	-0.005 (-0.04, 0.03)	-0.004 (-0.04, 0.04)	0.003 (-0.03, 0.03)	0.003 (-0.03, 0.04)	-0.02 (-0.1, 0.06)	-0.01 (-0.09, 0.07)	0.002 (-0.04, 0.04)	0.002 (-0.04, 0.05)	-0.01 (-0.06, 0.04)	-0.01 (-0.06, 0.04)
Q3	-0.01 (-0.06, 0.04)	-0.003 (-0.05, 0.05)	-0.02 (-0.05, 0.02)	-0.01 (-0.05, 0.02)	-0.01 (-0.04, 0.03)	-0.005 (-0.05, 0.04)	-0.02 (-0.1, 0.07)	-0.004 (-0.09, 0.08)	-0.01 (-0.04, 0.02)	-0.01 (-0.04, 0.02)	-0.01 (-0.05, 0.03)	-0.01 (-0.05, 0.03)
Q4	-0.02 (-0.07, 0.02)	-0.02 (-0.07, 0.03)	-0.005 (-0.04, 0.03)	-0.003 (-0.04, 0.03)	-0.01 (-0.05, 0.03)	-0.01 (-0.05, 0.03)	-0.03 (-0.11, 0.06)	-0.02 (-0.11, 0.07)	0.01 (-0.03, 0.04)	0.002 (-0.03, 0.04)	0.03 (-0.01, 0.07)	0.03 (-0.01, 0.07)
Q5	ref.	ref.	ref.	ref.	ref.	ref.	ref.	ref.	ref.	ref.	ref.	ref.
P trend	0.25	0.24	0.07	0.06	0.07	0.11	0.12	0.12	0.01	0.02	0.19	0.24
**Acrophase**												
Q1	0.02 (-0.03, 0.06)	0.01 (-0.04, 0.06)	0.01 (-0.01, 0.04)	0.01 (-0.01, 0.04)	0.02 (-0.03, 0.06)	0.01 (-0.04, 0.06)	0.02 (-0.07, 0.1)	0.01 (-0.07, 0.09)	-0.001 (-0.03, 0.03)	0.003 (-0.03, 0.04)	0.01 (-0.04, 0.06)	0.02 (-0.04, 0.07)
Q2	0.01 (-0.04, 0.06)	0.01 (-0.04, 0.06)	0.01 (-0.03, 0.05)	0.01 (-0.04, 0.05)	-0.001 (-0.04, 0.04)	-0.002 (-0.04, 0.04)	-0.001 (-0.1, 0.1)	-0.01 (-0.11, 0.09)	-0.01 (-0.04, 0.02)	-0.01 (-0.04, 0.02)	-0.01 (-0.07, 0.05)	-0.01 (-0.07, 0.05)
Q3	ref.	ref.	ref.	ref.	ref.	ref.	ref.	ref.	ref.	ref.	ref.	ref.
Q4	-0.02 (-0.09, 0.04)	-0.02 (-0.09, 0.05)	-0.01 (-0.05, 0.02)	-0.01 (-0.05, 0.03)	-0.01 (-0.06, 0.03)	-0.02 (-0.06, 0.03)	-0.03 (-0.13, 0.07)	-0.03 (-0.13, 0.07)	-0.01 (-0.05, 0.03)	-0.01 (-0.05, 0.03)	-0.02 (-0.07, 0.03)	-0.02 (-0.07, 0.03)
Q5	0.04 (-0.02, 0.1)	0.03 (-0.03, 0.09)	0.02 (-0.02, 0.06)	0.02 (-0.03, 0.06)	0.04 (0.003, 0.07)	0.03 (-0.003, 0.07)	0.05 (-0.06, 0.16)	0.05 (-0.06, 0.15)	-0.02 (-0.07, 0.02)	-0.02 (-0.07, 0.03)	0.002 (-0.06, 0.06)	0.005 (-0.06, 0.07)
P trend	0.98	0.84	0.53	0.55	0.59	0.62	0.73	0.54	0.35	0.35	0.55	0.51
**IS**												
Q1	0.01 (-0.05, 0.07)	0.004 (-0.06, 0.07)	0.02 (-0.02, 0.07)	0.03 (-0.03, 0.08)	0.06 (0.02, 0.09)	0.05 (0.02, 0.09)	0.03 (-0.05, 0.12)	0.03 (-0.06, 0.13)	-0.08 (-0.12, -0.04)	-0.07 (-0.11, -0.03)	-0.03 (-0.1, 0.04)	-0.02 (-0.09, 0.05)
Q2	0.01 (-0.06, 0.08)	-0.005 (-0.08, 0.07)	0.02 (-0.03, 0.07)	0.02 (-0.03, 0.07)	0.03 (-0.005, 0.07)	0.03 (-0.01, 0.06)	0.05 (-0.03, 0.13)	0.04 (-0.05, 0.12)	-0.04 (-0.09, 0.002)	-0.03 (-0.07, 0.01)	-0.01 (-0.05, 0.03)	-0.001 (-0.05, 0.04)
Q3	-0.02 (-0.06, 0.03)	-0.02 (-0.07, 0.03)	-0.01 (-0.05, 0.03)	-0.01 (-0.05, 0.04)	0.01 (-0.03, 0.05)	0.004 (-0.04, 0.05)	0.01 (-0.06, 0.08)	0.01 (-0.06, 0.08)	-0.07 (-0.11, -0.02)	-0.06 (-0.1, -0.02)	-0.01 (-0.05, 0.04)	-0.001 (-0.05, 0.04)
Q4	-0.04 (-0.09, 0.01)	-0.03 (-0.08, 0.01)	-0.02 (-0.06, 0.02)	-0.01 (-0.05, 0.02)	0.02 (-0.03, 0.06)	0.02 (-0.03, 0.06)	-0.03 (-0.1, 0.03)	-0.03 (-0.09, 0.04)	-0.01 (-0.04, 0.02)	-0.01 (-0.04, 0.02)	-0.03 (-0.07, 0.02)	-0.02 (-0.07, 0.03)
Q5	ref.	ref.	ref.	ref.	ref.	ref.	ref.	ref.	ref.	ref.	ref.	ref.
P trend	0.37	0.60	0.10	0.11	0.003	0.01	0.09	0.18	0.001	0.002	0.46	0.75
**IV**												
Q1	ref.	ref.	ref.	ref.	ref.	ref.	ref.	ref.	ref.	ref.	ref.	ref.
Q2	0.004 (-0.05, 0.06)	0.01 (-0.05, 0.07)	-0.01 (-0.05, 0.02)	-0.01 (-0.05, 0.03)	-0.004 (-0.05, 0.04)	-0.002 (-0.04, 0.04)	-0.02 (-0.09, 0.05)	-0.01 (-0.08, 0.06)	-0.01 (-0.04, 0.03)	-0.01 (-0.04, 0.03)	0.02 (-0.03, 0.07)	0.02 (-0.03, 0.07)
Q3	0.02 (-0.03, 0.07)	0.02 (-0.03, 0.07)	0.01 (-0.04, 0.05)	0.01 (-0.04, 0.05)	0.001 (-0.05, 0.05)	-0.00 (-0.05, 0.05)	-0.02 (-0.1, 0.06)	-0.01 (-0.09, 0.07)	-0.002 (-0.03, 0.03)	-0.004 (-0.03, 0.03)	0.03 (-0.03, 0.09)	0.02 (-0.04, 0.08)
Q4	0.04 (-0.02, 0.1)	0.04 (-0.02, 0.09)	0.02 (-0.02, 0.07)	0.03 (-0.02, 0.07)	-0.02 (-0.07, 0.02)	-0.02 (-0.07, 0.03)	0.03 (-0.03, 0.1)	0.04 (-0.02, 0.09)	-0.04 (-0.08, 0.004)	-0.04 (-0.08, 0.005)	0.02 (-0.05, 0.09)	0.02 (-0.05, 0.1)
Q5	0.01 (-0.04, 0.07)	0.02 (-0.03, 0.07)	0.03 (-0.02, 0.08)	0.03 (-0.02, 0.08)	0.01 (-0.02, 0.04)	0.01 (-0.02, 0.04)	0.01 (-0.05, 0.08)	0.03 (-0.05, 0.1)	-0.03 (-0.07, 0.01)	-0.03 (-0.06, 0.01)	0.02 (-0.04, 0.08)	0.03 (-0.03, 0.09)
P trend	0.25	0.18	0.07	0.06	0.98	1.00	0.23	0.18	0.045	0.04	0.46	0.36

Regression results presented as log-transformed liver enzyme levels.

Model 3: adjusted for age, gender, race and ethnicity, education, household income, marital status, smoking, alcohol, hepatitis B, hepatitis C, hepatitis E and diabetes status.

Model 4: adjusted for age, gender, race and ethnicity, education, household income, marital status, smoking, alcohol, hepatitis B, hepatitis C, hepatitis E and BMI.

Abbreviations: NHANES, National Health and Nutrition Examination Survey; ALT, alanine aminotransferase; AST, aspartate aminotransferase; ALP, alkaline phosphatase; GGT, gamma glutamyl transaminase; CI, confidence interval; IS, Interdaily stability; IV, Intradaily variability

Table S4. Associations (OR and 95% CI) between quintiles of rest-activity rhythm characteristics and abnormal levels of ALT, AST, ALP, GGT, albumin and bilirubin, adjusting for physical activity and sleeping duration, NHANES 2011 – 2014

	ALT	AST	ALP	GGT	Albumin	Bilirubin
**F statistic**						
Q1	1.02 (0.57, 1.84)	1.41 (0.87, 2.27)	1.84 (0.70, 4.81)	1.78 (1.06, 2.97)	3.61 (1.38, 9.46)	1.78 (0.93, 3.43)
Q2	1.31 (0.79, 2.16)	1.34 (0.90, 1.99)	1.15 (0.42, 3.15)	1.68 (0.94, 2.98)	1.78 (0.63, 4.97)	1.05 (0.44, 2.52)
Q3	1.00 (0.69, 1.45)	0.81 (0.52, 1.27)	0.81 (0.43, 1.52)	1.04 (0.68, 1.59)	1 (0.32, 3.06)	1.26 (0.57, 2.77)
Q4	0.79 (0.47, 1.31)	0.90 (0.55, 1.48)	0.31 (0.10, 0.92)	1.05 (0.68, 1.63)	1.36 (0.47, 3.91)	0.9 (0.4, 2.04)
Q5	ref.	ref.	ref.	ref.	ref.	ref.
P trend	0.26	0.04	0.04	0.02	0.02	0.26
**Amplitude**						
Q1	1.18 (0.67, 2.07)	1.50 (0.81, 2.77)	2.03 (0.61, 6.75)	1.50 (0.88, 2.57)	1.5 (0.49, 4.59)	1.92 (0.81, 4.57)
Q2	1.34 (0.81, 2.22)	1.17 (0.70, 1.96)	1.27 (0.46, 3.52)	1.28 (0.81, 2.03)	0.76 (0.26, 2.19)	0.95 (0.28, 3.23)
Q3	1.01 (0.59, 1.75)	1.12 (0.65, 1.91)	0.73 (0.25, 2.17)	1.10 (0.65, 1.85)	0.51 (0.16, 1.65)	1.96 (0.74, 5.15)
Q4	0.89 (0.46, 1.74)	0.80 (0.40, 1.62)	2.04 (0.84, 4.91)	1.10 (0.63, 1.91)	0.61 (0.21, 1.78)	2.03 (0.83, 4.96)
Q5	ref.	ref.	ref.	ref.	ref.	ref.
P trend	0.11	0.06	0.49	0.05	0.4	0.047
**Mesor**						
Q1	1.12 (0.66, 1.89)	1.23 (0.65, 2.32)	1.09 (0.43, 2.74)	0.94 (0.50, 1.75)	0.81 (0.22, 3.03)	1.41 (0.54, 3.72)
Q2	0.91 (0.54, 1.54)	0.84 (0.45, 1.57)	0.58 (0.23, 1.45)	0.71 (0.44, 1.12)	1.03 (0.27, 3.89)	1.39 (0.57, 3.39)
Q3	1.26 (0.86, 1.84)	1.27 (0.77, 2.11)	0.58 (0.29, 1.15)	0.96 (0.58, 1.61)	1.18 (0.49, 2.84)	0.56 (0.21, 1.44)
Q4	1.08 (0.71, 1.64)	1.13 (0.65, 1.96)	0.47 (0.19, 1.14)	0.84 (0.56, 1.26)	0.98 (0.38, 2.5)	1.19 (0.49, 2.9)
Q5	ref.	ref.	ref.	ref.	ref.	ref.
P trend	0.98	0.89	0.66	0.62	0.5	0.97
**Amplitude:Mesor**						
Q1	1.34 (0.84, 2.13)	1.65 (1.05, 2.60)	1.73 (0.69, 4.37)	1.50 (0.95, 2.39)	1.29 (0.5, 3.34)	1.72 (0.71, 4.21)
Q2	1.12 (0.72, 1.73)	0.94 (0.59, 1.52)	1.58 (0.74, 3.39)	0.81 (0.51, 1.27)	0.52 (0.21, 1.31)	1.28 (0.45, 3.63)
Q3	1.01 (0.61, 1.67)	0.88 (0.53, 1.45)	1.12 (0.44, 2.84)	0.99 (0.63, 1.57)	0.55 (0.22, 1.39)	1.56 (0.68, 3.57)
Q4	0.98 (0.58, 1.67)	1.00 (0.66, 1.52)	1.31 (0.46, 3.68)	0.88 (0.53, 1.47)	0.45 (0.2, 0.99)	2.08 (0.74, 5.85)
Q5	ref.	ref.	ref.	ref.	ref.	ref.
P trend	0.12	0.07	0.15	0.09	0.33	0.12
**Acrophase**						
Q1	ref.	ref.	ref.	ref.	ref.	ref.
Q2	1.02 (0.64, 1.63)	0.99 (0.57, 1.72)	1.1 (0.54, 2.24)	0.98 (0.61, 1.58)	1.02 (0.44, 2.36)	0.96 (0.36, 2.55)
Q3	0.89 (0.55, 1.44)	0.95 (0.61, 1.46)	0.68 (0.28, 1.65)	0.95 (0.54, 1.67)	1.38 (0.55, 3.48)	1.94 (0.79, 4.76)
Q4	0.93 (0.59, 1.46)	0.97 (0.63, 1.50)	0.64 (0.31, 1.34)	0.86 (0.52, 1.41)	1.21 (0.51, 2.85)	1.3 (0.51, 3.32)
Q5	0.91 (0.55, 1.53)	1.06 (0.65, 1.74)	0.93 (0.54, 1.60)	1.14 (0.72, 1.79)	1.52 (0.75, 3.1)	1.73 (0.71, 4.18)
P trend	0.55	0.84	0.20	0.82	0.43	0.03
**Interdaily stability**						
Q1	0.77 (0.44, 1.35)	1.35 (0.82, 2.21)	2.21 (0.96, 5.11)	1.52 (0.91, 2.55)	3.63 (1.27, 10.35)	1.63 (0.72, 3.69)
Q2	0.93 (0.51, 1.68)	1.28 (0.75, 2.18)	1.06 (0.42, 2.7)	1.35 (0.79, 2.32)	1.53 (0.46, 5.06)	2.64 (0.95, 7.29)

Q3	0.8 (0.48, 1.34)	0.97 (0.58, 1.62)	1.08 (0.49, 2.37)	1.04 (0.66, 1.63)	2.51 (0.79, 7.99)	1.75 (0.82, 3.74)
Q4	0.72 (0.48, 1.08)	0.79 (0.55, 1.15)	1.1 (0.45, 2.72)	0.95 (0.59, 1.53)	1.67 (0.5, 5.52)	1.23 (0.5, 3.01)
Q5	ref.	ref.	ref.	ref.	ref.	ref.
P trend	0.63	0.08	0.11	0.047	0.006	0.03
**Intradaily variability**						
Q1	ref.	ref.	ref.	ref.	ref.	ref.
Q2	0.9 (0.52, 1.56)	0.88 (0.51, 1.51)	0.76 (0.34, 1.73)	0.92 (0.64, 1.34)	1.41 (0.48, 4.15)	2.41 (0.62, 9.32)
Q3	1.01 (0.57, 1.8)	1.05 (0.58, 1.89)	1.1 (0.49, 2.44)	0.91 (0.59, 1.41)	1.14 (0.36, 3.57)	2.88 (0.87, 9.51)
Q4	1.17 (0.66, 2.09)	1.01 (0.54, 1.89)	0.61 (0.26, 1.44)	1.13 (0.8, 1.6)	2.49 (0.72, 8.66)	5.49 (1.25, 24.01)
Q5	0.84 (0.53, 1.32)	1.36 (0.72, 2.56)	1.98 (0.72, 5.46)	1.11 (0.69, 1.79)	2.26 (0.74, 6.91)	3.04 (0.68, 13.61)
P trend	0.93	0.26	0.18	0.37	0.03	0.02

Abnormal levels defined as >47 IU/L in men or >30 IU/L in women for ALT, >33 IU/L for AST, >113 IU/L for ALP, >65 IU/L in men or >36 IU/L in women for GGT, <3.7 g/dL for albumin, and >1.3 mg/dL for bilirubin

Model adjusted for age, gender, race and ethnicity, education, household income, marital status, smoking, alcohol, hepatitis B, hepatitis C, hepatitis E, physical activity and sleeping duration

Abbreviations: NHANES, National Health and Nutrition Examination Survey; ALT, alanine aminotransferase; AST, aspartate aminotransferase; ALP, alkaline phosphatase; GGT, gamma glutamyl transaminase

Table S5. Associations (OR and 95% CI) between quintiles of F statistic and abnormal levels of ALT, AST, ALP, GGT, albumin and bilirubin, after removing night shift worker proxied by L5MD, NHANES 2011 – 2014

	F Statistic					
**Abnormal levels of liver enzymes**	**Q1**	**Q2**	**Q3**	**Q4**	**Q5**	**P trend**
**F statistic, median (IQR)**	189 (140, 230)	325 (294, 354)	453 (421, 481)	599 (560, 651)	851 (764, 999)	
**ALT**						
**N (%)**[b]	128 (11)	136 (12)	136 (11)	99 (8.1)	122 (9.8)	
**OR (95% CI)**						
**Model 1**	1.22 (0.84, 1.79)	1.3 (0.89, 1.89)	1.12 (0.83, 1.52)	0.82 (0.56, 1.19)	ref.	0.04
**Model 2**	1.2 (0.71, 2.02)	1.37 (0.83, 2.24)	1.12 (0.76, 1.64)	0.85 (0.53, 1.36)	ref.	0.12
**Model 3**	1.08 (0.62, 1.86)	1.26 (0.75, 2.12)	1.05 (0.69, 1.58)	0.82 (0.51, 1.3)	ref.	0.27
**Model 4**	1.04 (0.58, 1.87)	1.23 (0.74, 2.05)	1.02 (0.68, 1.55)	0.81 (0.49, 1.32)	ref.	0.34
**AST**						
**N (%)**[b]	157 (14)	149 (11)	111 (8.2)	100 (8.4)	102 (8.7)	
**OR (95% CI)**						
**Model 1**	1.6 (1.18, 2.16)	1.3 (0.98, 1.73)	0.9 (0.66, 1.21)	0.93 (0.68, 1.28)	ref.	0.001
**Model 2**	1.44 (0.92, 2.25)	1.35 (0.93, 1.95)	0.84 (0.57, 1.24)	0.92 (0.6, 1.4)	ref.	0.04
**Model 3**	1.31 (0.83, 2.08)	1.27 (0.86, 1.87)	0.8 (0.53, 1.19)	0.89 (0.59, 1.36)	ref.	0.09
**Model 4**	1.38 (0.83, 2.27)	1.3 (0.88, 1.93)	0.82 (0.54, 1.24)	0.91 (0.58, 1.41)	ref.	0.07
**ALP**						
**N (%)**[b]	65 (5.9)	43 (3.6)	30 (2.2)	19 (0.8)	30 (2.2)	
**OR (95% CI)**						
**Model 1**	3.16 (1.9, 5.25)	1.74 (0.88, 3.43)	1.09 (0.72, 1.67)	0.37 (0.17, 0.8)	ref.	<0.001
**Model 2**	2.55 (1.48, 4.41)	1.53 (0.72, 3.24)	1.02 (0.61, 1.7)	0.32 (0.11, 0.93)	ref.	<0.001
**Model 3**	2.24 (1.27, 3.94)	1.39 (0.64, 3)	0.95 (0.57, 1.61)	0.3 (0.1, 0.89)	ref.	0.001
**Model 4**	2.44 (1.31, 4.55)	1.39 (0.63, 3.09)	0.95 (0.54, 1.67)	0.25 (0.08, 0.77)	ref.	0.001
**GGT**						
**N (%)**[b]	160 (14)	143 (12)	127 (7.9)	103 (7.5)	86 (6.8)	
**OR (95% CI)**						
**Model 1**	2.5 (1.76, 3.55)	2.02 (1.32, 3.09)	1.29 (0.94, 1.77)	1.16 (0.82, 1.64)	ref.	<0.001
**Model 2**	2.31 (1.44, 3.72)	1.99 (1.15, 3.43)	1.2 (0.81, 1.78)	1.17 (0.77, 1.78)	ref.	0.002
**Model 3**	2.08 (1.3, 3.33)	1.83 (1.04, 3.22)	1.13 (0.75, 1.7)	1.12 (0.74, 1.69)	ref.	0.004
**Model 4**	1.97 (1.15, 3.35)	1.75 (0.97, 3.18)	1.07 (0.7, 1.66)	1.1 (0.71, 1.7)	ref.	0.01
**Albumin**						
**N (%)**[b]	73 (5.5)	39 (2.9)	29 (1.5)	26 (2)	11 (1.1)	
**OR (95% CI)**						
**Model 1**	6.33 (2.94, 13.67)	2.96 (1.35, 6.51)	1.56 (0.65, 3.71)	2.02 (0.86, 4.71)	ref.	<0.001
**Model 2**	4.45 (1.76, 11.23)	2.35 (0.95, 5.85)	1.17 (0.41, 3.3)	1.4 (0.52, 3.78)	ref.	0.002
**Model 3**	3.99 (1.54, 10.33)	2.12 (0.82, 5.48)	1.08 (0.36, 3.24)	1.34 (0.49, 3.62)	ref.	0.003
**Model 4**	3.71 (1.33, 10.32)	1.87 (0.68, 5.17)	0.99 (0.32, 3.1)	1.25 (0.43, 3.64)	ref.	0.01
**Bilirubin**						

**N (%)**[b]	45 (4.4)	24 (2.3)	31 (3)	33 (2.5)	30 (2.6)	
**OR (95% CI)**						
**Model 1**	1.48 (0.9, 2.42)	0.83 (0.4, 1.72)	1.04 (0.56, 1.94)	0.92 (0.48, 1.76)	ref.	0.29
**Model 2**	1.55 (0.83, 2.89)	0.84 (0.37, 1.89)	1.04 (0.51, 2.12)	0.8 (0.37, 1.69)	ref.	0.25
**Model 3**	1.52 (0.8, 2.9)	0.83 (0.37, 1.87)	1.02 (0.49, 2.14)	0.79 (0.36, 1.73)	ref.	0.27
**Model 4**	1.65 (0.83, 3.28)	0.89 (0.39, 2.05)	1.1 (0.5, 2.45)	0.84 (0.36, 1.94)	ref.	0.19

Abnormal levels defined as >47 IU/L in men or >30 IU/L in women for ALT, >33 IU/L for AST, >113 IU/L for ALP, >65 IU/L in men or >36 IU/L in women for GGT, <3.7 g/dL for albumin, and >1.3 mg/dL for bilirubin

[b]Percentage weighted using sample weights.

Model 1: adjusted for age and gender.

Model 2: adjusted for age, gender, race and ethnicity, education, household income, marital status, smoking, alcohol, hepatitis B, hepatitis C and hepatitis E.

Model 3: adjusted for age, gender, race and ethnicity, education, household income, marital status, smoking, alcohol, hepatitis B, hepatitis C, hepatitis E and diabetes status.

Model 4: adjusted for age, gender, race and ethnicity, education, household income, marital status, smoking, alcohol, hepatitis B, hepatitis C, hepatitis E and BMI.

Abbreviations: NHANES, National Health and Nutrition Examination Survey; ALT, alanine aminotransferase; AST, aspartate aminotransferase; ALP, alkaline phosphatase; GGT, gamma glutamyl transaminase; IQR, interquartile range; CI, confidence interval; OR, odds ratio

Table S6. Associations (OR and 95% CI) between quintiles of rest-activity rhythm characteristics and abnormal levels of ALT, AST, ALP and GGT, stratified by alcohol drinking status, NHANES 2011 – 2014

	ALT		AST		ALP		GGT	
	Never/former/light	Moderate/heavy	Never/former/light	Moderate/heavy	Never/former/light	Moderate/heavy	Never/former/light	Moderate/heavy
**F statistic**								
Q1	1.22 (0.73, 2.04)	1.19 (0.45, 3.11)	1.34 (0.79, 2.25)	1.66 (0.77, 3.55)	1.84 (1.11, 3.05)	7.61 (1.54, 37.73)	2.36 (1.51, 3.68)	1.67 (0.65, 4.26)
Q2	1.53 (0.91, 2.58)	1.18 (0.45, 3.06)	1.25 (0.82, 1.9)	1.48 (0.75, 2.91)	1.33 (0.66, 2.7)	0.56 (0.05, 6.4)	1.97 (1.18, 3.29)	1.74 (0.67, 4.51)
Q3	1.13 (0.72, 1.75)	0.98 (0.52, 1.84)	0.81 (0.47, 1.4)	0.83 (0.43, 1.6)	0.79 (0.42, 1.48)	1.97 (0.19, 20.63)	1.14 (0.8, 1.63)	1.17 (0.56, 2.48)
Q4	1.03 (0.67, 1.58)	0.34 (0.13, 0.88)	1.06 (0.67, 1.67)	0.5 (0.23, 1.08)	0.33 (0.12, 0.9)	0.29 (0.01, 6.69)	1.42 (1,004 2.02)	0.61 (0.26, 1.42)
Q5	ref.	ref.	ref.	ref.	ref.	ref.	ref.	ref.
$P_{interaction}$	0.35		0.24		0.32		0.52	
**Amplitude**								
Q1	1.2 (0.76, 1.91)	1.48 (0.55, 3.93)	1.17 (0.65, 2.09)	2.05 (0.81, 5.21)	4.01 (2.21, 7.28)	1.21 (0.17, 8.68)	1.72 (1.08, 2.73)	1.99 (0.71, 5.54)
Q2	1.34 (0.95, 1.88)	1.9 (0.6, 5.97)	0.94 (0.6, 1.46)	1.64 (0.6, 4.46)	2.28 (1.14, 4.53)	0.63 (0.1, 4.14)	1.16 (0.75, 1.8)	2.5 (0.97, 6.45)
Q3	1.1 (0.71, 1.7)	0.93 (0.38, 2.31)	0.9 (0.58, 1.4)	1.65 (0.57, 4.76)	1.4 (0.51, 3.83)	0.15 (0.02, 1.37)	1.19 (0.7, 2.02)	1.16 (0.35, 3.85)
Q4	0.92 (0.51, 1.65)	0.82 (0.26, 2.57)	0.78 (0.42, 1.45)	0.8 (0.32, 1.98)	3.88 (1.88, 7.98)	0.06 (0.004, 0.87)	1.21 (0.76, 1.95)	0.93 (0.33, 2.64)
Q5	ref.	ref.	ref.	ref.	ref.	ref.	ref.	ref.
$P_{interaction}$	0.30		0.19		0.68		0.26	
**Mesor**								
Q1	1.42 (0.85, 2.39)	0.99 (0.36, 2.71)	1.17 (0.74, 1.85)	1.32 (0.52, 3.37)	1.53 (0.82, 2.84)	1.46 (0.25, 8.44)	1.47 (0.91, 2.37)	1.12 (0.49, 2.59)
Q2	0.96 (0.51, 1.81)	1.44 (0.6, 3.5)	0.71 (0.41, 1.26)	1.27 (0.61, 2.64)	0.65 (0.26, 1.65)	0.75 (0.08, 6.79)	0.91 (0.61, 1.36)	0.96 (0.38, 2.43)
Q3	1.28 (0.85, 1.93)	1.8 (0.79, 4.11)	1.24 (0.77, 1.99)	1.36 (0.71, 2.63)	0.76 (0.39, 1.48)	0.11 (0.01, 0.84)	1.15 (0.72, 1.83)	1.21 (0.55, 2.69)
Q4	1.05 (0.7, 1.57)	1.4 (0.54, 3.67)	1.11 (0.68, 1.81)	1.11 (0.49, 2.47)	0.44 (0.18, 1.09)	0.77 (0.14, 4.14)	0.92 (0.57, 1.51)	0.92 (0.5, 1.68)
Q5	ref.	ref.	ref.	ref.	ref.	ref.	ref.	ref.
$P_{interaction}$	0.70		0.67		0.52		0.36	
**Amplitude:Mesor**								
Q1	1.2 (0.72, 2.01)	2.49 (0.995, 6.24)	1.38 (0.86, 2.23)	2.64 (1.13, 6.18)	2.6 (1.03, 6.59)	1.75 (0.31, 9.95)	1.43 (0.81, 2.54)	2.8 (1.17, 6.7)
Q2	1.17 (0.76, 1.78)	1.2 (0.42, 3.38)	0.93 (0.57, 1.52)	0.96 (0.32, 2.94)	3.08 (1.61, 5.88)	0.07 (0.01, 1)	0.81 (0.49, 1.32)	1.24 (0.48, 3.2)
Q3	1.04 (0.64, 1.69)	0.84 (0.37, 1.93)	0.75 (0.48, 1.18)	1.2 (0.49, 2.91)	1.92 (0.75, 4.91)	0.2 (0.03, 1.36)	1.03 (0.66, 1.62)	0.93 (0.31, 2.76)
Q4	1 (0.62, 1.62)	1.11 (0.31, 4)	1.07 (0.69, 1.66)	0.67 (0.23, 1.96)	2.47 (0.85, 7.17)	0 (0, 0)	0.95 (0.55, 1.66)	0.96 (0.3, 3.02)
Q5	ref.	ref.	ref.	ref.	ref.	ref.	ref.	ref.
$P_{interaction}$	0.18		0.17		0.84		0.10	
**Acrophase**								
Q1	ref.	ref.	ref.	ref.	ref.	ref.	ref.	ref.
Q2	0.94 (0.57, 1.55)	1.31 (0.43, 3.96)	1.14 (0.64, 2.02)	0.7 (0.25, 1.92)	1.06 (0.51, 2.19)	1.35 (0.32, 5.68)	1.11 (0.66, 1.87)	0.61 (0.3, 1.23)
Q3	0.81 (0.48, 1.36)	1.44 (0.53, 3.88)	0.92 (0.5, 1.71)	0.97 (0.32, 3)	0.6 (0.25, 1.44)	1.04 (0.11, 9.63)	0.92 (0.55, 1.53)	0.95 (0.4, 2.23)
Q4	0.91 (0.57, 1.45)	1.13 (0.4, 3.21)	1.2 (0.75, 1.94)	0.53 (0.19, 1.49)	0.78 (0.38, 1.63)	0 (0, 0)	0.97 (0.6, 1.55)	0.75 (0.34, 1.68)
Q5	0.87 (0.51, 1.47)	1.33 (0.59, 3.01)	1.11 (0.67, 1.85)	1 (0.34, 2.94)	1.01 (0.63, 1.62)	0.75 (0.14, 4.13)	1.19 (0.75, 1.91)	1.27 (0.57, 2.79)
$P_{interaction}$	0.45		0.66		0.59		0.51	
**Interdaily stability**								
Q1	0.83 (0.49, 1.4)	1.17 (0.47, 2.94)	1.29 (0.77, 2.16)	1.71 (0.63, 4.65)	2.5 (1.44, 4.34)	4.8 (1.02, 22.67)	2 (1.19, 3.36)	1.58 (0.7, 3.57)
Q2	0.95 (0.52, 1.74)	1.27 (0.42, 3.84)	1.27 (0.69, 2.33)	1.33 (0.67, 2.65)	1.17 (0.58, 2.35)	1.59 (0.16, 15.32)	1.36 (0.88, 2.11)	2.04 (0.89, 4.71)
Q3	0.9 (0.56, 1.47)	0.86 (0.3, 2.47)	1.04 (0.62, 1.74)	0.84 (0.3, 2.4)	1.16 (0.66, 2.05)	1.5 (0.22, 10)	1.17 (0.8, 1.7)	1.31 (0.7, 2.45)
Q4	0.8 (0.53, 1.21)	0.55 (0.2, 1.54)	0.8 (0.5, 1.26)	0.79 (0.28, 2.24)	1.41 (0.6, 3.34)	0.14 (0.01, 1.81)	1.07 (0.69, 1.65)	0.91 (0.43, 1.94)
Q5	ref.	ref.	ref.	ref.	ref.	ref.	ref.	ref.
$P_{interaction}$	0.20		0.88		0.30		0.69	
**Intradaily variability**								
Q1	ref.	ref.	ref.	ref.	ref.	ref.	ref.	ref.
Q2	1.03 (0.63, 1.7)	0.82 (0.36, 1.85)	0.76 (0.4, 1.46)	1.15 (0.58, 2.29)	0.8 (0.38, 1.69)	0.74 (0.1, 5.32)	1.25 (0.81, 1.92)	0.69 (0.34, 1.43)
Q3	1.07 (0.69, 1.65)	1.38 (0.49, 3.92)	0.9 (0.54, 1.49)	1.35 (0.43, 4.19)	1.24 (0.51, 3)	0.57 (0.17, 1.94)	1.28 (0.82, 1.99)	0.72 (0.3, 1.74)
Q4	1.23 (0.78, 1.93)	1.95 (0.7, 5.45)	0.83 (0.49, 1.38)	1.53 (0.53, 4.49)	0.56 (0.28, 1.13)	1.59 (0.28, 8.85)	1.3 (0.81, 2.08)	1.81 (0.95, 3.45)

Q5	0.92 (0.62, 1.35)	1.54 (0.54, 4.43)	1.29 (0.74, 2.23)	1.29 (0.52, 3.18)	2.33 (1.22, 4.46)	2.9 (0.55, 15.17)	1.56 (1.03, 2.36)	1.37 (0.47, 3.98)
$P_{interaction}$	0.42		0.90		0.62		0.86	

Abnormal levels defined as >47 IU/L in men or >30 IU/L in women for ALT, >33 IU/L in men and women for AST, >113 IU/L in men and women for ALP and >65 IU/L in men or >36 IU/L in women for GGT
Model adjusted for age, gender, race and ethnicity, education, household income, marital status, smoking, alcohol, hepatitis B, hepatitis C and hepatitis E.
Light drinker were classified as having average daily drinking volume of me <1 for men and <0.5 for women; moderate drinker as 1 to 2 for men and 0.5 to 1 for women; heavy drinker as ≥2 for men and ≥1 for women.
Abbreviations: NHANES, National Health and Nutrition Examination Survey; ALT, alanine aminotransferase; AST, aspartate aminotransferase; ALP, alkaline phosphatase; GGT, gamma glutamyl transaminase

Table S7. Associations (OR and 95% CI) between quintiles of rest-activity rhythm characteristics and abnormal levels of ALT, AST, ALP and GGT, stratified by BMI, NHANES 2011 – 2014

	ALT			AST			ALP			GGT		
	<25	25-30	>=30	<25	25-30	>=30	<25	25-30	>=30	<25	25-30	>=30
**F statistic**												
Q1	2.04 (0.79, 5.29)	0.96 (0.34, 2.72)	0.87 (0.47, 1.61)	2.04 (0.67, 6.2)	1 (0.49, 2.01)	1.31 (0.76, 2.24)	12.37 (1.95, 78.61)	1.11 (0.4, 3.06)	1.96 (0.85, 4.52)	1.61 (0.57, 4.57)	1.59 (0.92, 2.77)	1.54 (0.77, 3.08)
Q2	0.96 (0.4, 2.26)	1.02 (0.44, 2.38)	1.59 (0.91, 2.78)	1.33 (0.54, 3.27)	0.88 (0.49, 1.57)	1.45 (0.85, 2.48)	5.69 (1.35, 23.98)	0.3 (0.11, 0.83)	1.6 (0.5, 5.08)	1.29 (0.59, 2.86)	0.96 (0.45, 2.02)	1.93 (0.97, 3.83)
Q3	1.56 (0.71, 3.45)	1.23 (0.67, 2.27)	0.75 (0.43, 1.31)	1.08 (0.42, 2.81)	0.7 (0.39, 1.24)	0.77 (0.44, 1.34)	2.84 (0.8, 10.11)	0.98 (0.46, 2.08)	0.39 (0.13, 1.17)	0.96 (0.46, 2.03)	1.15 (0.61, 2.15)	0.84 (0.48, 1.49)
Q4	0.69 (0.23, 2.05)	0.59 (0.26, 1.3)	0.9 (0.5, 1.61)	0.67 (0.23, 1.95)	0.8 (0.39, 1.64)	0.88 (0.46, 1.68)	1.29 (0.27, 6.14)	0.21 (0.05, 0.82)	0.23 (0.06, 0.84)	0.96 (0.31, 2.97)	0.83 (0.33, 2.09)	1.14 (0.7, 1.84)
Q5	ref.	ref.	ref.	ref.	ref.	ref.	ref.	ref.	ref.	ref.	ref.	ref.
$P_{interaction}$	0.59			0.70			0.65			0.74		
**Amplitude**												
Q1	1.14 (0.39, 3.32)	1.31 (0.64, 2.69)	0.95 (0.56, 1.59)	1.27 (0.5, 3.26)	1.01 (0.5, 2.04)	1.24 (0.58, 2.66)	1.6 (0.28, 9.08)	3.06 (0.48, 19.35)	1.89 (0.98, 3.63)	1.34 (0.47, 3.8)	0.9 (0.46, 1.76)	1.79 (0.99, 3.22)
Q2	1.45 (0.58, 3.62)	1.69 (0.77, 3.69)	1.14 (0.76, 1.72)	0.9 (0.38, 2.13)	1.62 (0.77, 3.38)	0.84 (0.44, 1.62)	0.43 (0.06, 2.97)	4.82 (0.99, 23.41)	0.64 (0.21, 1.98)	1.47 (0.51, 4.24)	1.11 (0.57, 2.13)	1.27 (0.68, 2.39)
Q3	0.79 (0.33, 1.92)	1.18 (0.58, 2.4)	0.87 (0.46, 1.65)	0.8 (0.3, 2.12)	1.6 (0.82, 3.14)	0.74 (0.36, 1.5)	0.25 (0.05, 1.29)	1.18 (0.22, 6.43)	0.6 (0.2, 1.78)	1.21 (0.42, 3.51)	0.73 (0.32, 1.68)	1.27 (0.71, 2.25)
Q4	0.75 (0.29, 1.93)	1.06 (0.5, 2.22)	0.72 (0.36, 1.43)	0.66 (0.29, 1.49)	1.24 (0.58, 2.67)	0.49 (0.21, 1.17)	0.64 (0.07, 6.25)	4.38 (0.97, 19.72)	1.38 (0.52, 3.63)	1.64 (0.6, 4.47)	0.67 (0.29, 1.54)	1.13 (0.58, 2.21)
Q5	ref.	ref.	ref.	ref.	ref.	ref.	ref.	ref.	ref.	ref.	ref.	ref.
$P_{interaction}$	0.27			0.72			0.94			0.53		
**Mesor**												
Q1	1.35 (0.5, 3.61)	1.62 (0.82, 3.22)	0.96 (0.57, 1.6)	1.1 (0.47, 2.55)	1.4 (0.77, 2.52)	1.01 (0.65, 1.58)	9.95 (1.84, 53.63)	1.05 (0.35, 3.18)	1.07 (0.51, 2.22)	0.71 (0.32, 1.55)	1.21 (0.56, 2.62)	0.98 (0.52, 1.84)
Q2	1.11 (0.38, 3.25)	0.82 (0.34, 2)	1.04 (0.61, 1.75)	1 (0.5, 1.99)	0.71 (0.33, 1.54)	0.85 (0.42, 1.71)	5.39 (0.63, 46.4)	0.32 (0.08, 1.29)	0.45 (0.18, 1.15)	0.87 (0.32, 2.35)	0.48 (0.22, 1.04)	0.83 (0.48, 1.45)
Q3	0.99 (0.38, 2.56)	1.85 (0.95, 3.6)	1.13 (0.67, 1.92)	0.92 (0.32, 2.65)	1.57 (0.83, 2.96)	1.17 (0.54, 2.5)	2.84 (0.44, 18.29)	0.83 (0.24, 2.8)	0.37 (0.17, 0.79)	0.71 (0.22, 2.22)	1.21 (0.57, 2.59)	1.03 (0.58, 1.84)
Q4	1.06 (0.32, 3.46)	1.35 (0.72, 2.56)	0.88 (0.5, 1.56)	1.17 (0.54, 2.54)	1.23 (0.58, 2.58)	1.02 (0.5, 2.1)	2.49 (0.43, 14.5)	0.71 (0.2, 2.55)	0.31 (0.12, 0.84)	0.88 (0.28, 2.79)	0.84 (0.41, 1.74)	0.87 (0.55, 1.37)
Q5	ref.	ref.	ref.	ref.	ref.	ref.	ref.	ref.	ref.	ref.	ref.	ref.
$P_{interaction}$	0.57			0.64			0.36			0.63		
**Amplitude:Mesor**												
Q1	1.46 (0.55, 3.85)	1.69 (0.86, 3.34)	0.92 (0.53, 1.59)	1.56 (0.73, 3.32)	1.7 (0.74, 3.89)	1.32 (0.68, 2.57)	1.13 (0.21, 6.06)	6.36 (0.85, 47.63)	1.32 (0.51, 3.44)	1.28 (0.48, 3.42)	1.52 (0.73, 3.15)	1.66 (0.82, 3.38)
Q2	0.87 (0.32, 2.31)	1.18 (0.57, 2.46)	0.92 (0.53, 1.59)	0.68 (0.31, 1.5)	1.24 (0.59, 2.61)	0.78 (0.39, 1.58)	0.54 (0.09, 3.31)	6.72 (0.93, 48.67)	1.18 (0.42, 3.27)	1.03 (0.36, 3.01)	0.65 (0.3, 1.4)	0.98 (0.52, 1.86)
Q3	1.07 (0.5, 2.32)	0.92 (0.42, 2.04)	0.92 (0.53, 1.59)	0.82 (0.42, 1.61)	0.99 (0.41, 2.4)	0.78 (0.38, 1.61)	0.34 (0.05, 2.19)	2.91 (0.4, 21.23)	1.06 (0.41, 2.72)	1.43 (0.55, 3.71)	0.7 (0.33, 1.47)	1.04 (0.57, 1.89)
Q4	0.84 (0.3, 2.36)	1.36 (0.62, 2.98)	0.92 (0.53, 1.59)	0.67 (0.24, 1.91)	1.71 (0.87, 3.33)	0.67 (0.37, 1.19)	0.48 (0.05, 4.91)	4.04 (0.51, 31.97)	1.23 (0.34, 4.48)	0.75 (0.22, 2.54)	1.11 (0.57, 2.14)	0.84 (0.39, 1.84)
Q5	ref.	ref.	ref.	ref.	ref.	ref.	ref.	ref.	ref.	ref.	ref.	ref.
$P_{interaction}$	0.47			0.63			0.62			0.66		
**Acrophase**												
Q1	ref.	ref.	ref.	ref.	ref.	ref.	ref.	ref.	ref.	ref.	ref.	ref.
Q2	0.48 (0.21, 1.13)	0.82 (0.38, 1.78)	1.48 (0.9, 2.45)	0.56 (0.25, 1.25)	0.74 (0.31, 1.78)	1.67 (0.97, 2.89)	0.28 (0.08, 1)	1.08 (0.41, 2.79)	1.56 (0.56, 4.36)	0.69 (0.28, 1.7)	0.59 (0.3, 1.17)	1.45 (0.89, 2.35)
Q3	0.36 (0.15, 0.87)	0.98 (0.49, 1.95)	1.34 (0.67, 2.65)	0.27 (0.12, 0.6)	0.86 (0.39, 1.92)	2 (1.04, 3.85)	0.88 (0.23, 3.36)	0.29 (0.09, 0.97)	1.05 (0.33, 3.29)	0.75 (0.33, 1.69)	1.19 (0.52, 2.69)	0.87 (0.5, 1.49)
Q4	0.63 (0.28, 1.43)	0.93 (0.49, 1.8)	1.45 (0.81, 2.62)	0.44 (0.18, 1.1)	0.98 (0.47, 2.02)	1.8 (0.86, 3.79)	0.86 (0.23, 3.21)	0.49 (0.15, 1.66)	0.69 (0.3, 1.62)	0.55 (0.2, 1.49)	0.51 (0.2, 1.33)	1.37 (0.87, 2.16)
Q5	0.69 (0.23, 2.12)	0.85 (0.36, 2)	1.2 (0.67, 2.15)	0.71 (0.28, 1.82)	0.93 (0.47, 1.84)	1.68 (0.84, 3.36)	0.65 (0.25, 1.67)	0.6 (0.27, 1.36)	1.34 (0.54, 3.28)	0.51 (0.22, 1.19)	1.41 (0.67, 2.95)	1.49 (0.96, 2.3)
$P_{interaction}$	0.31			0.15			0.44			0.04		
**Interdaily stability**												
Q1	1.2 (0.41, 3.57)	0.8 (0.33, 1.97)	0.79 (0.4, 1.57)	2.98 (0.99, 8.96)	0.9 (0.41, 1.94)	1.33 (0.71, 2.5)	10.38 (1.8, 59.99)	1.25 (0.38, 4.11)	4.64 (1.57, 13.72)	0.83 (0.4, 1.73)	1.6 (0.84, 3.06)	1.71 (0.86, 3.41)
Q2	1.34 (0.55, 3.27)	0.85 (0.39, 1.88)	1 (0.51, 1.97)	1.77 (0.78, 4.05)	0.92 (0.49, 1.74)	1.28 (0.64, 2.54)	7.52 (0.86, 66.07)	0.25 (0.09, 0.66)	2.52 (0.65, 9.84)	1.08 (0.43, 2.73)	1.3 (0.56, 3.04)	1.34 (0.78, 2.29)
Q3	0.51 (0.19, 1.32)	0.75 (0.35, 1.62)	1.02 (0.62, 1.67)	1.47 (0.49, 4.42)	0.71 (0.36, 1.41)	0.96 (0.51, 1.82)	5.71 (1.18, 27.66)	0.44 (0.17, 1.12)	1.86 (0.53, 6.57)	1 (0.41, 2.43)	0.8 (0.4, 1.59)	1.18 (0.7, 1.97)
Q4	0.72 (0.26, 2.01)	0.65 (0.33, 1.29)	0.84 (0.51, 1.38)	0.84 (0.33, 2.15)	0.64 (0.32, 1.28)	0.99 (0.64, 1.53)	1.39 (0.24, 7.9)	0.49 (0.15, 1.55)	2.55 (0.74, 8.78)	0.32 (0.12, 0.9)	0.98 (0.54, 1.76)	1.25 (0.66, 2.34)
Q5	ref.	ref.	ref.	ref.	ref.	ref.	ref.	ref.	ref.	ref.	ref.	ref.
$P_{interaction}$	0.94			0.28			0.61			0.68		
**Intradaily variability**												
Q1	ref.	ref.	ref.	ref.	ref.	ref.	ref.	ref.	ref.	ref.	ref.	ref.
Q2	0.97 (0.32, 2.99)	1.53 (0.66, 3.55)	0.68 (0.38, 1.22)	0.74 (0.33, 1.67)	1.04 (0.46, 2.33)	0.57 (0.28, 1.16)	0.38 (0.12, 1.26)	0.72 (0.2, 2.55)	1.05 (0.33, 3.27)	1.28 (0.51, 3.23)	1.22 (0.52, 2.86)	0.64 (0.4, 1.05)
Q3	1.66 (0.69, 3.98)	1.3 (0.62, 2.75)	0.99 (0.55, 1.79)	0.85 (0.36, 2.01)	0.94 (0.48, 1.82)	1.04 (0.57, 1.91)	0.83 (0.22, 3.2)	0.74 (0.18, 3.08)	1.21 (0.64, 2.28)	1.12 (0.62, 2.02)	0.83 (0.41, 1.67)	1.05 (0.6, 1.84)
Q4	2.16 (0.91, 5.11)	1.53 (0.77, 3.06)	0.97 (0.55, 1.71)	1.23 (0.55, 2.73)	0.93 (0.5, 1.73)	0.74 (0.37, 1.47)	2.16 (0.44, 10.53)	0.4 (0.13, 1.31)	0.66 (0.21, 2.09)	1.55 (0.8, 3)	1.15 (0.54, 2.44)	1.06 (0.6, 1.89)

Q5	1.51 (0.51, 4.43)	1.38 (0.63, 3.04)	0.68 (0.38, 1.23)	2 (0.89, 4.52)	1.11 (0.6, 2.06)	0.85 (0.41, 1.76)	4.13 (1, 17.05)	1.34 (0.44, 4.05)	2.82 (1.13, 7.03)	0.84 (0.36, 1.95)	1 (0.52, 1.94)	1.24 (0.65, 2.37)
$P_{interaction}$	0.06			0.01			0.63			0.34		

Abnormal levels defined as >47 IU/L in men or >30 IU/L in women for ALT, >33 IU/L in men and women for AST, >113 IU/L in men and women for ALP and >65 IU/L in men or >36 IU/L in women for GGT
Model adjusted for age, gender, race and ethnicity, education, household income, marital status, smoking, alcohol, hepatitis B, hepatitis C and hepatitis E.
Abbreviations: NHANES, National Health and Nutrition Examination Survey; ALT, alanine aminotransferase; AST, aspartate aminotransferase; ALP, alkaline phosphatase; GGT, gamma glutamyl transaminase

Table S8. Associations (OR and 95% CI) between rest-activity rhythm characteristics quintiles and abnormal levels of ALT, AST, ALP and GGT, stratified by diabetes status, NHANES 2011 – 2014

	ALT		AST		ALP		GGT	
	With diabetes	Without diabetes	With diabetes	Without diabetes	With diabetes	Without diabetes	With diabetes	Without diabetes
**F statistic**								
Q1	0.68 (0.3, 1.57)	1.11 (0.65, 1.88)	2.01 (0.95, 4.29)	1.07 (0.68, 1.67)	1.72 (0.54, 5.49)	2.32 (1.15, 4.68)	2.93 (1.39, 6.17)	1.43 (0.86, 2.37)
Q2	0.97 (0.38, 2.51)	1.32 (0.79, 2.21)	1.63 (0.73, 3.63)	1.02 (0.69, 1.52)	1.71 (0.4, 7.32)	1.02 (0.41, 2.55)	2.11 (1.09, 4.11)	1.52 (0.91, 2.51)
Q3	0.73 (0.22, 2.46)	1.08 (0.72, 1.62)	1.21 (0.52, 2.81)	0.68 (0.43, 1.08)	0.62 (0.2, 1.95)	0.99 (0.49, 2.01)	2.03 (0.8, 5.12)	0.9 (0.58, 1.39)
Q4	0.45 (0.19, 1.09)	0.82 (0.5, 1.35)	0.72 (0.3, 1.77)	0.84 (0.56, 1.26)	0.32 (0.04, 2.32)	0.36 (0.14, 0.9)	1.52 (0.68, 3.39)	0.96 (0.64, 1.46)
Q5	ref.	ref.	ref.	ref.	ref.	ref.	ref.	ref.
$P_{interaction}$	0.50		0.18		0.70		0.26	
**Amplitude**								
Q1	1.13 (0.36, 3.54)	0.98 (0.62, 1.54)	2.43 (0.77, 7.7)	0.89 (0.53, 1.48)	2.59 (1.02, 6.53)	2.24 (1, 5.02)	1.94 (1.05, 3.58)	1.25 (0.79, 1.99)
Q2	1.14 (0.39, 3.34)	1.3 (0.79, 2.15)	1.87 (0.73, 4.81)	0.88 (0.55, 1.39)	1.21 (0.29, 5.11)	1.39 (0.57, 3.37)	1.13 (0.56, 2.28)	1.34 (0.89, 2.01)
Q3	0.78 (0.2, 3.08)	0.96 (0.65, 1.41)	1.63 (0.44, 5.96)	0.9 (0.59, 1.37)	1.04 (0.35, 3.05)	0.54 (0.2, 1.48)	0.79 (0.29, 2.17)	1.14 (0.69, 1.89)
Q4	0.63 (0.15, 2.76)	0.87 (0.48, 1.56)	0.69 (0.13, 3.63)	0.76 (0.45, 1.29)	3.1 (1.09, 8.83)	1.55 (0.69, 3.5)	0.79 (0.31, 2.02)	1.11 (0.66, 1.88)
Q5	ref.	ref.	ref.	ref.	ref.	ref.	ref.	ref.
$P_{interaction}$	0.73		0.01		0.56		0.21	
**Mesor**								
Q1	2.03 (0.89, 4.64)	1.05 (0.65, 1.68)	1.2 (0.55, 2.61)	1.04 (0.65, 1.69)	3.49 (1.22, 9.99)	0.94 (0.45, 1.96)	0.99 (0.38, 2.57)	1.01 (0.58, 1.73)
Q2	1.13 (0.43, 2.96)	1.01 (0.67, 1.52)	0.59 (0.21, 1.65)	0.89 (0.52, 1.52)	1.25 (0.35, 4.44)	0.42 (0.17, 1)	0.44 (0.18, 1.05)	0.85 (0.56, 1.3)
Q3	2.55 (1.14, 5.74)	1.13 (0.81, 1.58)	1.57 (0.51, 4.85)	1.12 (0.77, 1.64)	0.8 (0.22, 2.91)	0.58 (0.27, 1.25)	0.94 (0.46, 1.92)	1.05 (0.7, 1.57)
Q4	1.94 (0.8, 4.68)	0.94 (0.63, 1.38)	1.53 (0.6, 3.93)	1.04 (0.6, 1.78)	0.64 (0.16, 2.54)	0.48 (0.2, 1.17)	0.59 (0.25, 1.39)	0.94 (0.59, 1.49)
Q5	ref.	ref.	ref.	ref.	ref.	ref.	ref.	ref.
$P_{interaction}$	0.75		0.55		0.03		0.71	
**Amplitude:Mesor**								
Q1	1.12 (0.45, 2.79)	1.22 (0.77, 1.95)	2.96 (1.08, 8.13)	1.11 (0.65, 1.9)	1.3 (0.33, 5.18)	2.4 (1.01, 5.68)	1.45 (0.78, 2.68)	1.5 (0.94, 2.41)
Q2	1.05 (0.41, 2.65)	1.07 (0.7, 1.64)	1.63 (0.59, 4.55)	0.78 (0.48, 1.25)	1.47 (0.58, 3.71)	1.87 (0.84, 4.19)	0.72 (0.36, 1.43)	0.9 (0.58, 1.41)
Q3	0.92 (0.29, 2.95)	0.98 (0.63, 1.53)	1.38 (0.39, 4.92)	0.77 (0.48, 1.23)	1.15 (0.34, 3.86)	1.2 (0.53, 2.75)	0.6 (0.29, 1.24)	1.14 (0.74, 1.77)
Q4	0.66 (0.2, 2.25)	1.03 (0.67, 1.58)	1.44 (0.49, 4.23)	0.88 (0.62, 1.26)	1.86 (0.28, 12.55)	1.21 (0.43, 3.35)	0.48 (0.2, 1.2)	1.03 (0.64, 1.66)
Q5	ref.	ref.	ref.	ref.	ref.	ref.	ref.	ref.
$P_{interaction}$	0.69		0.01		0.05		0.40	
**Acrophase**								
Q1	ref.	ref.	ref.	ref.	ref.	ref.	ref.	ref.
Q2	1.15 (0.62, 2.12)	1.04 (0.62, 1.75)	1.85 (0.83, 4.1)	0.83 (0.47, 1.47)	1.02 (0.34, 3.07)	1.13 (0.52, 2.43)	1.81 (0.92, 3.55)	0.8 (0.48, 1.35)
Q3	0.73 (0.3, 1.74)	0.97 (0.57, 1.64)	1.54 (0.63, 3.76)	0.81 (0.51, 1.28)	1.26 (0.43, 3.65)	0.43 (0.17, 1.05)	1.53 (0.7, 3.35)	0.8 (0.49, 1.31)
Q4	1.1 (0.63, 1.9)	0.97 (0.59, 1.57)	1.81 (0.87, 3.75)	0.82 (0.53, 1.25)	0.81 (0.36, 1.85)	0.62 (0.24, 1.58)	1.44 (0.8, 2.6)	0.74 (0.44, 1.26)
Q5	0.51 (0.27, 0.93)	1.08 (0.61, 1.9)	0.76 (0.38, 1.52)	1.06 (0.64, 1.77)	1.21 (0.46, 3.2)	0.81 (0.47, 1.41)	1.67 (0.89, 3.15)	1.1 (0.69, 1.74)
$P_{interaction}$	0.66		0.99		0.13		0.01	
**Interdaily stability**								
Q1	0.48 (0.21, 1.08)	0.87 (0.52, 1.46)	1.31 (0.66, 2.57)	1.22 (0.77, 1.94)	5.63 (1.56, 20.37)	2.03 (1.05, 3.94)	2.27 (1.09, 4.74)	1.26 (0.79, 2.04)
Q2	0.6 (0.24, 1.51)	1.06 (0.59, 1.9)	1.59 (0.83, 3.05)	1.05 (0.64, 1.72)	2.66 (0.57, 12.49)	0.87 (0.35, 2.18)	1.24 (0.7, 2.2)	1.28 (0.81, 2.03)
Q3	1.1 (0.57, 2.11)	0.71 (0.41, 1.23)	1.42 (0.67, 2.99)	0.73 (0.44, 1.22)	4.7 (1.25, 17.66)	0.67 (0.29, 1.52)	1.51 (0.87, 2.62)	0.9 (0.59, 1.37)
Q4	0.56 (0.24, 1.29)	0.76 (0.49, 1.17)	0.74 (0.31, 1.74)	0.77 (0.49, 1.21)	3.02 (0.77, 11.86)	0.8 (0.32, 2.02)	0.66 (0.34, 1.3)	0.95 (0.6, 1.5)
Q5	ref.	ref.	ref.	ref.	ref.	ref.	ref.	ref.
$P_{interaction}$	0.39		0.59		0.16		0.09	
**Intradaily variability**								
Q1	ref.	ref.	ref.	ref.	ref.	ref.	ref.	ref.
Q2	1.04 (0.4, 2.68)	0.84 (0.52, 1.35)	0.74 (0.23, 2.38)	0.76 (0.48, 1.19)	5.84 (1.17, 29.09)	0.45 (0.2, 1.01)	1.52 (0.66, 3.5)	0.84 (0.64, 1.08)
Q3	1.05 (0.44, 2.48)	1.07 (0.61, 1.88)	0.88 (0.38, 2.02)	0.95 (0.55, 1.65)	4.59 (0.9, 23.45)	0.67 (0.28, 1.6)	1.48 (0.67, 3.28)	0.87 (0.59, 1.28)
Q4	1.19 (0.45, 3.13)	1.19 (0.66, 2.14)	1.04 (0.41, 2.66)	0.8 (0.44, 1.46)	2.72 (0.7, 10.65)	0.52 (0.21, 1.27)	1.4 (0.7, 2.81)	1.15 (0.84, 1.57)

Q5	0.76 (0.3, 1.93)	0.87 (0.55, 1.37)	1.04 (0.4, 2.7)	1.08 (0.7, 1.65)	10.89 (3.81, 31.11)	1.28 (0.62, 2.64)	2.56 (1.39, 4.71)	0.82 (0.54, 1.24)
$P_{interaction}$	0.73		0.58		0.04		0.04	

Abnormal levels defined as >47 IU/L in men or >30 IU/L in women for ALT, >33 IU/L in men and women for AST, >113 IU/L in men and women for ALP and >65 IU/L in men or >36 IU/L in women for GGT
Model adjusted for age, gender, race and ethnicity, education, household income, marital status, smoking, alcohol, hepatitis B, hepatitis C and hepatitis E.
Abbreviations: NHANES, National Health and Nutrition Examination Survey; ALT, alanine aminotransferase; AST, aspartate aminotransferase; ALP, alkaline phosphatase; GGT, gamma glutamyl transaminase

Table S9. Associations (OR and 95% CI) between rest-activity rhythm characteristics quintiles and abnormal levels of ALT, AST, ALP, and GGT, stratified by sessions attended, NHANES 2011 – 2014

	ALT			AST			ALP			GGT		
	Morning	Afternoon	Evening	Morning	Afternoon	Evening	Morning	Afternoon	Evening	Morning	Afternoon	Evening
**F statistic**												
Q1	1.36 (0.75, 2.47)	0.99 (0.48, 2.06)	0.78 (0.25, 2.47)	1.51 (0.81, 2.79)	1.45 (0.75, 2.82)	0.56 (0.2, 1.6)	2.15 (0.87, 5.28)	2.11 (0.98, 4.52)	4.03 (0.29, 56.67)	2.29 (1.34, 3.94)	1.64 (0.92, 2.94)	1.09 (0.25, 4.82)
Q2	1.73 (0.93, 3.19)	1.05 (0.56, 1.96)	0.98 (0.39, 2.49)	1.44 (0.76, 2.74)	1.07 (0.63, 1.82)	0.81 (0.36, 1.81)	1.69 (0.6, 4.8)	0.96 (0.38, 2.4)	1.16 (0.08, 16.99)	2.51 (1.38, 4.59)	0.86 (0.46, 1.62)	2.31 (0.52, 10.15)
Q3	0.89 (0.58, 1.35)	1.43 (0.78, 2.63)	1 (0.37, 2.69)	0.68 (0.36, 1.29)	1.09 (0.59, 2)	0.59 (0.25, 1.39)	0.83 (0.32, 2.14)	0.79 (0.34, 1.81)	1.89 (0.12, 28.65)	1.28 (0.73, 2.24)	0.95 (0.54, 1.69)	1.34 (0.31, 5.72)
Q4	0.64 (0.31, 1.31)	1.27 (0.65, 2.48)	0.39 (0.16, 1)	1.09 (0.44, 2.69)	0.92 (0.51, 1.66)	0.32 (0.11, 0.94)	0.37 (0.13, 1.07)	0.39 (0.12, 1.23)	0 (0, 0)	1.56 (0.86, 2.81)	0.69 (0.37, 1.28)	1.42 (0.47, 4.27)
Q5	ref.	ref.	ref.	ref.	ref.	ref.	ref.	ref.	ref.	ref.	ref.	ref.
$P_{interaction}$	0.16			0.32			0.24			0.52		
**Amplitude**												
Q1	1.36 (0.75, 2.49)	0.62 (0.33, 1.2)	2.65 (1, 7)	1.45 (0.82, 2.58)	0.94 (0.4, 2.18)	0.82 (0.37, 1.81)	2.12 (0.78, 5.75)	2.96 (0.92, 9.5)	3.13 (0.13, 77.53)	1.54 (0.85, 2.79)	1.86 (0.97, 3.57)	1.06 (0.32, 3.53)
Q2	1.2 (0.67, 2.15)	1.25 (0.67, 2.35)	3.28 (1.42, 7.61)	1.21 (0.72, 2.02)	0.89 (0.42, 1.92)	1.15 (0.36, 3.62)	1.3 (0.49, 3.44)	1.31 (0.34, 4.97)	8.15 (0.14, 485.77)	1.13 (0.62, 2.09)	1.57 (0.85, 2.89)	2.27 (0.79, 6.55)
Q3	0.96 (0.45, 2.05)	0.78 (0.45, 1.36)	2.05 (0.82, 5.13)	0.94 (0.53, 1.67)	1.12 (0.58, 2.18)	0.71 (0.28, 1.76)	0.69 (0.23, 2.11)	0.62 (0.14, 2.77)	4.64 (0.11, 202.84)	0.95 (0.56, 1.61)	1.52 (0.8, 2.88)	0.88 (0.22, 3.48)
Q4	0.75 (0.35, 1.64)	0.7 (0.37, 1.32)	2.14 (0.67, 6.87)	0.76 (0.34, 1.68)	0.6 (0.3, 1.21)	1.12 (0.42, 3)	1.87 (0.72, 4.86)	1.53 (0.35, 6.62)	17.31 (0.26, 1154.48)	1.04 (0.57, 1.88)	0.84 (0.45, 1.54)	2.23 (0.71, 7.01)
Q5	ref.	ref.	ref.	ref.	ref.	ref.	ref.	ref.	ref.	ref.	ref.	ref.
$P_{interaction}$	0.41			0.15			0.17			0.62		
**Mesor**												
Q1	1.69 (1.01, 2.83)	0.85 (0.52, 1.41)	1.28 (0.39, 4.2)	1.47 (0.82, 2.62)	0.94 (0.58, 1.51)	1.09 (0.34, 3.52)	1.85 (0.73, 4.72)	1.11 (0.66, 1.87)	1.74 (0.22, 14.03)	1.58 (0.98, 2.55)	0.93 (0.57, 1.54)	0.66 (0.18, 2.37)
Q2	1.29 (0.79, 2.11)	0.93 (0.45, 1.93)	0.69 (0.23, 2.05)	1.02 (0.54, 1.92)	0.76 (0.35, 1.66)	0.69 (0.23, 2.05)	0.69 (0.25, 1.89)	0.38 (0.1, 1.41)	2.8 (0.31, 25.01)	1.19 (0.71, 1.99)	0.64 (0.33, 1.24)	0.33 (0.09, 1.12)
Q3	2.02 (1.03, 3.93)	0.85 (0.49, 1.46)	1.24 (0.44, 3.5)	2.05 (1.07, 3.92)	0.83 (0.4, 1.7)	0.82 (0.2, 3.33)	0.48 (0.18, 1.28)	0.73 (0.29, 1.85)	0 (0, 0)	1.73 (0.82, 3.63)	0.6 (0.34, 1.06)	1.1 (0.32, 3.79)
Q4	1.48 (0.81, 2.72)	0.76 (0.46, 1.24)	0.95 (0.33, 2.71)	1.49 (0.88, 2.55)	1.05 (0.54, 2.03)	0.6 (0.19, 1.93)	0.63 (0.24, 1.69)	0.24 (0.08, 0.74)	4.78 (0.49, 46.83)	1.29 (0.69, 2.4)	0.62 (0.31, 1.24)	0.76 (0.25, 2.31)
Q5	ref.	ref.	ref.	ref.	ref.	ref.	ref.	ref.	ref.	ref.	ref.	ref.
$P_{interaction}$	0.65			0.30			0.15			0.63		
**Amplitude:Mesor**												
Q1	1.45 (0.96, 2.19)	0.97 (0.46, 2.03)	2.26 (0.98, 5.22)	1.63 (0.9, 2.96)	1.19 (0.53, 2.71)	1.48 (0.53, 4.16)	1.59 (0.51, 4.91)	3.1 (0.98, 9.8)	0.83 (0.11, 6.15)	1.4 (0.82, 2.38)	2.19 (1.13, 4.24)	1.71 (0.59, 5.01)
Q2	1.42 (0.91, 2.21)	0.83 (0.44, 1.59)	1.44 (0.73, 2.85)	1.41 (0.75, 2.67)	0.67 (0.33, 1.36)	0.36 (0.13, 1.02)	1.62 (0.6, 4.37)	2.23 (0.6, 8.35)	2.97 (0.18, 48.74)	0.9 (0.52, 1.56)	1.08 (0.56, 2.06)	0.84 (0.24, 2.88)
Q3	0.94 (0.52, 1.7)	0.82 (0.42, 1.61)	2.61 (1.35, 5.05)	0.78 (0.37, 1.66)	0.94 (0.44, 1.97)	0.96 (0.38, 2.43)	0.97 (0.36, 2.62)	1.61 (0.3, 8.65)	5.41 (0.5, 58.6)	0.92 (0.56, 1.52)	1.62 (0.88, 3.01)	0.53 (0.17, 1.66)
Q4	1.31 (0.78, 2.22)	0.71 (0.38, 1.35)	1.03 (0.33, 3.21)	1.16 (0.63, 2.14)	0.8 (0.39, 1.65)	0.93 (0.4, 2.13)	1.45 (0.35, 5.96)	1.56 (0.35, 6.91)	0.52 (0.03, 9.03)	0.91 (0.55, 1.5)	1.29 (0.59, 2.78)	0.47 (0.14, 1.61)
Q5	ref.	ref.	ref.	ref.	ref.	ref.	ref.	ref.	ref.	ref.	ref.	ref.
$P_{interaction}$	0.91			0.24			0.17			0.72		
**Acrophase**												
Q1	ref.	ref.	ref.	ref.	ref.	ref.	ref.	ref.	ref.	ref.	ref.	ref.
Q2	1 (0.61, 1.63)	0.97 (0.49, 1.91)	1.1 (0.52, 2.32)	0.77 (0.38, 1.56)	1.19 (0.6, 2.37)	1.28 (0.43, 3.84)	0.93 (0.36, 2.39)	1.61 (0.62, 4.2)	0.6 (0.18, 2.05)	1.3 (0.78, 2.17)	0.7 (0.31, 1.57)	0.4 (0.16, 1.02)
Q3	1.01 (0.58, 1.75)	0.87 (0.42, 1.8)	0.74 (0.24, 2.27)	0.8 (0.49, 1.32)	1.22 (0.59, 2.51)	0.81 (0.32, 2.11)	0.48 (0.21, 1.11)	1.14 (0.29, 4.42)	0.14 (0.01, 3.7)	0.79 (0.41, 1.54)	0.9 (0.47, 1.73)	1.43 (0.52, 3.94)
Q4	1.02 (0.64, 1.64)	0.91 (0.41, 2.04)	0.87 (0.27, 2.79)	0.94 (0.58, 1.53)	1.12 (0.54, 2.33)	0.84 (0.32, 2.22)	0.26 (0.11, 0.65)	1.28 (0.53, 3.13)	1.4 (0.22, 9)	0.95 (0.55, 1.65)	0.66 (0.3, 1.45)	1.23 (0.47, 3.2)
Q5	1.06 (0.62, 1.82)	0.81 (0.41, 1.6)	0.78 (0.25, 2.42)	0.92 (0.53, 1.59)	1.13 (0.54, 2.38)	1.12 (0.3, 4.23)	1.04 (0.55, 1.97)	0.93 (0.4, 2.15)	0.16 (0.01, 2.73)	1.18 (0.64, 2.16)	0.92 (0.57, 1.49)	2.66 (1.07, 6.62)
$P_{interaction}$	0.99			0.51			0.28			0.31		
**IS**												
Q1	0.99 (0.54, 1.81)	0.64 (0.32, 1.26)	0.9 (0.37, 2.21)	1.5 (0.85, 2.63)	1.04 (0.59, 1.82)	1.42 (0.55, 3.61)	3.33 (1.25, 8.88)	1.74 (0.82, 3.69)	8.83 (0.42, 185.35)	1.92 (1.15, 3.22)	1.48 (0.71, 3.11)	1.03 (0.24, 4.42)
Q2	1.01 (0.55, 1.86)	0.94 (0.51, 1.74)	1.03 (0.39, 2.73)	1.01 (0.59, 1.73)	1.47 (0.81, 2.66)	0.96 (0.43, 2.18)	1.02 (0.34, 3.03)	1.17 (0.47, 2.9)	1.29 (0.09, 18.38)	1.53 (0.87, 2.7)	0.97 (0.49, 1.89)	2.43 (0.59, 10.05)
Q3	0.8 (0.41, 1.54)	0.9 (0.44, 1.86)	0.76 (0.25, 2.28)	1.12 (0.56, 2.22)	0.75 (0.33, 1.7)	0.88 (0.34, 2.26)	1.69 (0.63, 4.51)	0.76 (0.27, 2.09)	0.53 (0.01, 26.62)	1.32 (0.81, 2.14)	0.84 (0.43, 1.61)	1.19 (0.28, 5.13)
Q4	0.73 (0.37, 1.47)	0.87 (0.44, 1.71)	0.78 (0.38, 1.57)	0.86 (0.5, 1.48)	0.79 (0.36, 1.74)	0.69 (0.25, 1.86)	1.45 (0.46, 4.61)	0.6 (0.24, 1.5)	5.78 (0.42, 78.72)	1.14 (0.55, 2.39)	0.83 (0.41, 1.67)	1.06 (0.32, 3.44)
Q5	ref.	ref.	ref.	ref.	ref.	ref.	ref.	ref.	ref.	ref.	ref.	ref.
$P_{interaction}$	0.95			0.55			0.20			0.65		
**IV**												
Q1	ref.	ref.	ref.	ref.	ref.	ref.	ref.	ref.	ref.	ref.	ref.	ref.
Q2	0.82 (0.37, 1.81)	0.81 (0.46, 1.43)	1.54 (0.67, 3.51)	0.93 (0.44, 1.98)	0.67 (0.39, 1.14)	0.74 (0.24, 2.29)	0.79 (0.29, 2.12)	0.9 (0.25, 3.27)	0.05 (0, 0.64)	0.77 (0.44, 1.36)	1.5 (0.86, 2.59)	0.57 (0.19, 1.67)
Q3	1.18 (0.56, 2.49)	0.99 (0.55, 1.8)	1.65 (0.65, 4.22)	1.05 (0.52, 2.12)	0.87 (0.48, 1.55)	1.31 (0.52, 3.29)	0.98 (0.49, 1.99)	1.06 (0.23, 4.85)	1.12 (0.08, 15.67)	0.94 (0.6, 1.49)	1.18 (0.69, 2.01)	0.87 (0.26, 2.92)
Q4	1.33 (0.61, 2.93)	0.99 (0.51, 1.91)	1.86 (0.86, 4.01)	0.89 (0.44, 1.8)	0.76 (0.41, 1.41)	1.53 (0.67, 3.51)	0.66 (0.25, 1.76)	0.83 (0.24, 2.86)	0.27 (0.03, 2.53)	0.97 (0.53, 1.77)	1.65 (0.98, 2.79)	1.16 (0.47, 2.85)

Q5	1.04 (0.55, 1.96)	0.65 (0.36, 1.15)	1.62 (0.7, 3.72)	1.07 (0.57, 1.99)	1.18 (0.63, 2.22)	1.22 (0.56, 2.65)	2.26 (0.89, 5.74)	2.8 (1.45, 5.42)	0.17 (0.01, 5.2)	1.16 (0.64, 2.12)	1.55 (0.86, 2.79)	0.62 (0.13, 2.9)
$P_{interaction}$	0.59			0.62			0.09			0.58		

Abnormal levels defined as >47 IU/L in men or >30 IU/L in women for ALT, >33 IU/L in men and women for AST, >113 IU/L in men and women for ALP and >65 IU/L in men or >36 IU/L in women for GGT

Model adjusted for age, gender, race and ethnicity, education, household income, marital status, smoking, alcohol, hepatitis B, hepatitis C and hepatitis E.

Abbreviations: NHANES, National Health and Nutrition Examination Survey; ALT, alanine aminotransferase; AST, aspartate aminotransferase; ALP, alkaline phosphatase; GGT, gamma glutamyl transaminase

Table S10. Associations (OR and 95% CI) between impaired rhythmicity score and abnormal levels of liver enzymes, albumin, and bilirubin, stratified by diabetes status, NHANES 2011 – 2014

Impaired rhythmicity score	ALT	AST	ALP	GGT	Albumin	Bilirubin
0	ref.	ref.	ref.	ref.	ref.	ref.
1	1.18 (0.79, 1.76)	1.15 (0.81, 1.62)	1.51 (0.75, 3.02)	1.38 (0.99, 1.92)	1.26 (0.66, 2.42)	0.91 (0.46, 1.8)
2	1.08 (0.72, 1.61)	1.31 (0.88, 1.95)	1.57 (0.79, 3.11)	1.75 (1.21, 2.52)	2.7 (1.57, 4.66)	1.35 (0.75, 2.43)
3+	1.22 (0.75, 2)	1.58 (0.88, 2.86)	3.77 (2.17, 6.56)	1.86 (1.19, 2.9)	4.65 (2.55, 8.46)	1.35 (0.7, 2.61)
P_{trend}	0.31	0.08	0.001	0.002	<0.001	0.21

Abnormal levels defined as >47 IU/L in men or >30 IU/L in women for ALT, >33 IU/L in men and women for AST, >113 IU/L in men and women for ALP and >65 IU/L in men or >36 IU/L in women for GGT
Model adjusted for age, gender, race and ethnicity, education, household income, marital status, smoking, alcohol, hepatitis B, hepatitis C and hepatitis E.
Abbreviations: NHANES, National Health and Nutrition Examination Survey; ALT, alanine aminotransferase; AST, aspartate aminotransferase; ALP, alkaline phosphatase; GGT, gamma glutamyl transaminase

Table S11. Associations (OR and 95% CI) between rest-activity rhythm characteristics quintiles and abnormal liver enzyme score, NHANES 2011 – 2014

	Abnormal liver enzyme score
**F statistic**	
Q1	1.7 (1.34, 2.17)
Q2	1.46 (1.18, 1.8)
Q3	0.98 (0.79, 1.2)
Q4	0.88 (0.71, 1.09)
Q5	ref.
P_{trend}	<0.001
**Amplitude**	
Q1	1.75 (1.28, 2.39)
Q2	1.3 (0.99, 1.71)
Q3	1.24 (0.98, 1.57)
Q4	1.15 (0.86, 1.54)
Q5	ref.
P_{trend}	0.72
**Mesor**	
Q1	1.37 (1.07, 1.76)
Q2	0.97 (0.77, 1.21)
Q3	1.1 (0.86, 1.42)
Q4	0.99 (0.78, 1.27)
Q5	ref.
P_{trend}	0.78
**Amplitude:Mesor**	
Q1	1.73 (1.27, 2.37)
Q2	1.11 (0.86, 1.44)
Q3	1.1 (0.84, 1.45)
Q4	1.18 (0.89, 1.56)
Q5	ref.
P_{trend}	0.14
**Acrophase**	
Q1	ref.
Q2	0.97 (0.78, 1.22)
Q3	1.03 (0.8, 1.32)
Q4	0.98 (0.78, 1.24)
Q5	1.14 (0.89, 1.47)
P_{trend}	0.36
**Interdaily stability**	
Q1	1.59 (1.18, 2.13)
Q2	1.34 (0.97, 1.85)
Q3	1.11 (0.85, 1.46)
Q4	0.92 (0.72, 1.18)
Q5	ref.
P_{trend}	<0.001
**Intradaily variability**	
Q1	ref.
Q2	0.95 (0.75, 1.21)

Q3	1.09 (0.82, 1.45)
Q4	1.5 (1.18, 1.92)
Q5	1.43 (1.16, 1.77)
P_{trend}	<0.001

Abnormal liver enzyme score represents the number of abnormal levels of ALT, ASLP, AST, GGT, albumin, and bilirubin, range from 0 to 3+.
Results obtained by ordered logistic regression.
Model adjusted for age, gender, race and ethnicity, education, household income, marital status, smoking, alcohol, hepatitis B, hepatitis C and hepatitis E.
Abbreviations: NHANES, National Health and Nutrition Examination Survey; ALT, alanine aminotransferase; AST, aspartate aminotransferase; ALP, alkaline phosphatase; GGT, gamma glutamyl transaminase
